# Disparate Interferon Signaling and Shared Aberrant Basaloid Cells in Single-Cell Profiling of Idiopathic Pulmonary Fibrosis and Systemic Sclerosis-Associated Interstitial Lung Disease

**DOI:** 10.3389/fimmu.2021.595811

**Published:** 2021-03-30

**Authors:** Eleanor Valenzi, Tracy Tabib, Anna Papazoglou, John Sembrat, Humberto E. Trejo Bittar, Mauricio Rojas, Robert Lafyatis

**Affiliations:** ^1^ Division of Pulmonary, Allergy and Critical Care Medicine, University of Pittsburgh, Pittsburgh, PA, United States; ^2^ Division of Rheumatology and Clinical Immunology, University of Pittsburgh, Pittsburgh, PA, United States; ^3^ Department of Pathology, University of Pittsburgh, Pittsburgh, PA, United States

**Keywords:** systemic sclerosis, systemic sclerosis (scleroderma), idiopathic pulmonary fibrosis, interstitial lung disease (ILD), single-cell RNA-sequencing (scRNA-seq)

## Abstract

Idiopathic pulmonary fibrosis (IPF) and systemic sclerosis-associated interstitial lung disease (SSc-ILD) differ in the predominant demographics and identified genetic risk alleles of effected patients, however both diseases frequently progress to respiratory failure and death. Contrasting advanced SSc-ILD to IPF provides insight to the role dysregulated immunity may play in pulmonary fibrosis. To analyze cell-type specific transcriptome commonalities and differences between IPF and SSc-ILD, we compared single-cell RNA-sequencing (scRNA-seq) of 21 explanted lung tissue specimens from patients with advanced IPF, SSc-ILD, and organ donor controls. Comparison of IPF and SSc-ILD tissue identified divergent patterns of interferon signaling, with interferon-gamma signaling upregulated in the *SPP1* ^hi^ and *FABP4* ^hi^ macrophages, cytotoxic T cells, and natural kill cells of IPF, while type I interferon signaling and production was upregulated in the corresponding SSc-ILD populations. Plasmacytoid dendritic cells were found in diseased lungs only, and exhibited upregulated cellular stress pathways in SSc-ILD compared to IPF. Alveolar type I cells were dramatically decreased in both IPF and SSc-ILD, with a distinct transcriptome signature separating these cells by disease. *KRT5^-^/KRT17^+^* aberrant basaloid cells exhibiting markers of cellular senescence and epithelial-mesenchymal transition were identified in SSc-ILD for the first time. In summary, our study utilizes the enriched capabilities of scRNA-seq to identify key divergent cell types and pathways between IPF and SSc-ILD, providing new insights into the shared and distinct mechanisms between idiopathic and autoimmune interstitial lung diseases.

## Introduction

Pulmonary fibrosis can occur as a consequence of autoimmunity, environmental exposures, or genetic mutations, as well as idiopathic disease. Idiopathic pulmonary fibrosis (IPF) is a progressive interstitial lung disease (ILD) of unknown etiology primarily occurring in the elderly, invariably progressing to respiratory failure, resulting in lung transplant or death. While some overlap exists between the clinical features, pathogenesis, and more recently the approved anti-fibrotic therapies for IPF and the connective tissue disease-associated ILDs, significant heterogeneity exists amongst the incidence and morbidity and mortality of the various autoimmune ILDs (1–3). In systemic sclerosis (SSc), an autoimmune disorder involving fibrosis and vasculopathy of the skin, lungs, and other organs, interstitial lung disease (ILD) occurs in up to 80% of patients, with 25-30% developing progressive disease resulting in respiratory failure (4). SSc predominantly occurs in women, although men with the disease have an increased risk for developing ILD, with an age of presentation between 30-55 years, while IPF predominantly occurs in men, with age of presentation between 60-75 years (5, 6). As it carries the highest rates of disease-associated mortality, primarily driven by pulmonary complications, examining advanced SSc-ILD in comparison to IPF presents a unique window into the role dysregulated immunity may play in pulmonary fibrosis (7, 8).

While the specific pathogenesis of both diseases remains unknown, current paradigms suggest that in IPF, repetitive epithelial cell microinjuries induce dysregulated restoration of the alveolar epithelium, activating a fibrotic response *via* the expansion of aberrant myofibroblasts, overproducing extracellular matrix (9). In SSc-ILD, activation of the innate and adaptive immune systems is hypothesized to result in endothelial and epithelial cell injury resulting in vasculopathy, aberrant transforming growth factor-beta (TGF-β) signaling, and the transformation to and expansion of myofibroblasts (10–12). In both diseases, resulting structural changes to the fibrotic lung parenchyma, including increasing tissue stiffness and hypoxia, generate a feed forward loop further promoting fibroblast activation and cellular injury (13–15). The significant role of inflammation is more established in the pathogenesis of SSc-ILD, with randomized clinical trials demonstrating modest therapeutic efficacy of the immunosuppressive agents cyclophosphamide and mycophenolate (16, 17). More recently, randomized trials in both Europe and the United States identified a mortality benefit for autologous stem cell transplantation in SSc, expanding therapeutic options for those with severe, progressive disease (18–20). Conversely, neither cyclophosphamide nor mycophenolate showed benefit in IPF clinical trials, and a trial of combined prednisone and azathioprine demonstrated increased mortality in those receiving immunosuppressive therapy (21–23).

The occurrence of pulmonary fibrosis within several rare genetic disorders, as well as the presence of familial pulmonary fibrosis have contributed to the increasing recognition that host genetic background influences the development and course of IPF and SSc-ILD for many patients (24). Rare variants in telomere-related (*TERT,TERC,PARN,RTEL1,DKC1,TINF2*) and surfactant-encoding genes (*SFTPC,SFTPA1,SFTPA2*), along with single nucleotide polymorphisms (SNPs) identified by genome-wide association studies (GWAS) in 20 independent loci currently account for 25-30% of the sporadic IPF disease risk (25, 26). The genes associated with these loci implicate a key role for telomere integrity, cell adhesion, and fibrogenic and host defense pathways in the pathogenesis of IPF. In SSc, a 2019 GWAS of 10,000 European patients identified 23 independent loci associated with SSc, increasing the total known SSc risk loci to 28 (27). The majority of gene products associated with these SSc risk loci are related to inflammation and autoimmunity, underscoring the immune etiology of SSc (28). Surprisingly, despite the similarities between the two diseases, there is no current overlap between the genetic variants or the identified HLA alleles associated with IPF and SSc-ILD. This is in contrast to ILD associated with rheumatoid arthritis (RA-ILD). The common *rs35705950* polymorphism in the promoter of MUC5B is associated with an increased risk of developing IPF as well as RA-ILD with a usual interstitial pneumonia (UIP) pattern, but not SSc-ILD (29, 30).

While the current paradigm for pathogenesis and genetic contribution to IPF and SSc-ILD differ, both diseases lead to a final common pathway of respiratory failure resulting in lung transplant or death. Investigating the shared and disparate mechanisms between SSc-ILD and IPF may yield important new insights influencing therapeutic development for both diseases. Previous microarray and bulk RNA-sequencing technologies are limited by their inherent averaging of gene expression across multiple disparate cell types. To examine transcriptomic commonalities and differences between advanced IPF and SSc-ILD, we compared single-cell RNA-sequencing (scRNA-seq) of peripheral parenchymal lung tissue obtained at the time of lung transplant from patients with IPF and SSc-ILD, and control lung tissue from organ donors without pre-existing lung disease. Comparison of IPF and SSc-ILD tissue identified divergent patterns of interferon signaling in IPF and SSc-ILD, increased cellular stress pathways in the plasmacytoid dendritic cells of SSc-ILD, a profound loss of alveolar type 1 (AT1) cells in both IPF and SSc-ILD with a distinct transcriptome signature defining each AT1 subset by disease, as well as identified aberrant basaloid cells in SSc-ILD for the first time.

## Materials and Methods

The University of Pittsburgh Institutional Review Board approved procedures involving human samples. Explanted subpleural peripheral lung tissue was digested and scRNA-sequencing performed as previously described (**Supplementary File 1**) (31, 32). All samples were processed in the same manner using 10X Genomics 3’ v2 chemistry reagents. Raw data was demultiplexed using Cell Ranger 3.0.2 mkfastq function and aligned to the human reference genome GRCh38. Data analysis was performed with the R package Seurat V3.1.1 and R V3.6.0 (33, 34). Cells were filtered for greater than 200 genes, less than 50,000 unique molecular identifiers (UMI), and less than 15 percent mitochondrial genes. To reduce batch effects in analyzing multiple samples, all control, IPF, and SSc-ILD samples were combined into 3 objects by disease status using Seurat’s IntegrateData function, followed by integration of these 3 objects (35). Following clustering and visualization with uniform manifold approximation and projection (UMAP) (36), cell populations were classified using multiple gene markers in the transcriptome. Doublet cells were manually identified as expressing markers of multiple cell types with elevated UMI counts and subsequently removed. Cell cycle phase was predicted using Seurat’s CellCycleScoring function. To better define individual cell types, the myeloid, lymphoid, epithelial, and stromal populations were then separately reclustered. Differential gene expression analysis for IPF versus SSc-ILD cells for each cluster was performed using the Wilcoxon rank-sum statistical test. Differences in mean proportions of cells comprising each cell type were compared with the non-parametric Kruskal-Wallis test with Dunn’s multiple comparison test. P-values less than 0.05 were considered to be statistically significant. Differentially expressed genes with a p-value less than 0.05 and absolute value natural log fold change greater than 0.5 were used as input for pathway enrichment analyses. Enriched gene ontology biological processes with a false discovery rate less than 5 percent were identified using the Gene Ontology Resource (37, 38). Hierarchical tree clustering of the epithelial cells was performed on gene average expression levels by Euclidean distance using Cluster 3.0, filtering out genes expressed at an average expression level less than 0.2 in fewer than 3 clusters (39).

## Results

### Study Population

In order to determine commonalities and differences between IPF and SSc-ILD, we performed single-cell RNA-sequencing (scRNA-seq) on 21 peripheral lung tissue specimens obtained at the time of lung transplant from patients with IPF (n=8 samples) and SSc-ILD (n=8) and control lung tissue from organ donors without pre-existing lung disease whose lungs were declined for transplant (n=5). Separate upper and lower lobe samples were included for each IPF and SSc-ILD patient and from one control patient, with only lower lobe samples available from the other 3 controls. For these analyses we added 2 not previously analyzed IPF samples with previously described samples (31, 32). The histopathology of adjacent lung tissue and clinical information for all samples was reviewed (**Table 1**, **Supplemental Figure 1**). All of the IPF and 7 of the SSc-ILD samples showed usual interstitial pneumonia (UIP) on histology, with varied amounts of diffuse alveolar damage, lymphoid aggregates, and myointimal thickening of the pulmonary arteries. The remaining SSc-ILD sample exhibited nonspecific interstitial pneumonia (NSIP) with acute lung injury. Although NSIP is traditionally regarded as the most common histopathology pattern in SSc-ILD (40), the high prevalence of UIP within our samples is consistent with the histopathology of most patients at the time of end-stage SSc-ILD resulting in respiratory failure (41).

**Table 1:** Characteristics of Patient Samples Demographics, the number of cells analyzed after filtering in the scRNA-seq analysis, pathological review of adjacent tissue, and clinical characteristics of the patient samples included.

Variable	IPF	SSc-ILD	Control
**N**	8	8	5
**Cells post-filtering, mean (SD)**	3995.88 [1031]	4039.63 [609.48]	4521.4 [959.79]
**Age (years), mean (SD)**	68.75 [1.09]	56.75 [9.5]	35.2 [17.09]
**Male, n (%)**	4 (50%)	6 (75%)	2 (40%)
**FEV1 (L), mean (SD)**	1.29 [0.66]	2.03 [0.51]	
**FEV1 predicted %, mean (SD)**	50.25 [19.21]	60.75 [11.32]	
**FVC (L), mean (SD)**	1.46 [0.81]	2.70 [0.88]	
**FVC predicted %, mean (SD)**	39.25 [14.45]	58.25 [11.54]	
**DLCO (ml), mean (SD)**	7.34 [3.34]	6.15 [3.33]	
**DLCO predicted %, mean (SD)**	33.75[13.74]	20.25 [9.23]	
**Mean PAP (mmHg), mean (SD)**	20.25 [6.91]	39.75 [12.58]	
**Specific therapies:** **Mycophenolate, n (%)** **Rituximab, n (%)** **Cyclophosphamide, n (%)** **Prednisone, n (%)** **Pirfenidone, n(%)**	4 (50%)	4 (50%) 2 (25%) 2 (25%) 2 (25%)	
**Usual interstitial pneumonia on explant pathology, n (%)**	8 (100%)	7 (87.5%)	

FEV1: forced expiratory volume in 1 second; FVC: forced vital capacity; DLCO: diffusion capacity of the lungs for carbon monoxide; PAP: pulmonary artery pressure.

Mean pulmonary artery pressure (mPAP) measurement is from the last right heart catheterization preceding lung transplantation. Immunosuppression listed includes the medications received in the 90 days preceding lung transplantation. Data presented as means (standard deviation [SD]]) or N (%).

**Figure 1:**
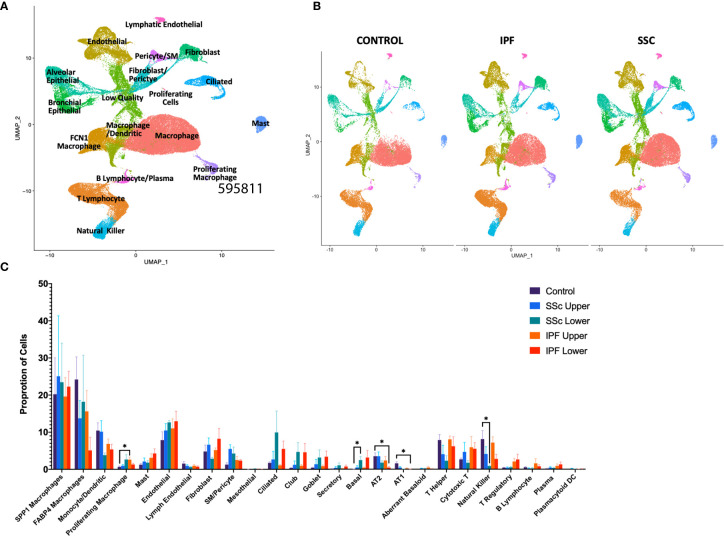
ScRNA-seq analysis of 8 IPF, 8 SSc-ILD, and 5 organ donor control lung samples. **(A)** UMAP plot of all 21 integrated samples, identified by cell type. **(B)** UMAP plots of all samples, divided by disease status. **(C)** Mean percentage of total cells comprised of each cell type, comparing, control, upper lobe IPF, lower lobe IPF, upper lobe SSc-ILD, and lower lobe SSc-ILD. Bars represent the mean percentage of total cells, with error bars representing the standard error of the mean. *=p-value<0.05. scRNA-seq, single-cell RNA-sequencing; IPF, idiopathic pulmonary fibrosis; SSc-ILD, systemic sclerosis-associated interstitial lung disease; UMAP, uniform manifold approximation and projection.

In total, we analyzed 85,756 cells, with individual cell types identified by multiple distinct markers (**Figure 1A**, **Supplemental Figure 2A** and **3**) and samples well-integrated by disease status and sample (**Figure 1B**, **Supplemental Figure 2B**). Myeloid, lymphoid, epithelial, and stromal populations were separately reclustered to identify more distinct cell phenotypes and allow for differential gene expression comparisons between IPF and SSc-ILD for all cell types. Evaluating the proportion of total cells present in each population by sample and disease status revealed many similar trends in the shifts of cell populations between control and fibrotic lungs (**Figure 1C**). Ciliated, club, goblet, basal, and the intermediate secretory cells all trended towards increased frequency in the fibrotic lower lobes, compared to the fibrotic upper lobes and control samples, reflecting the basal and mixed bronchial epithelial populations lining honeycomb cysts (42). Basal cells in SSc-ILD lower lobes were significantly increased in comparison to control lungs (p=0.0266). Alveolar type 1 and alveolar type 2 cells both trended towards a graded decrease, with the most dramatic loss of alveolar type 1 cells (p=0.0230) and alveolar type 2 cells (p=0.0365) occurring in the IPF lower lobes in comparison to controls. Amongst the lymphoid populations, natural killer cells were decreased in IPF and SSc-ILD compared to controls, with a significant change between controls and SSc-ILD lower lobes (p=0.0379). T regulatory, plasma, and plasmacytoid dendritic cells trended towards increase in IPF and SSc-ILD compared to controls. The smooth muscle and pericyte population was increased amongst the SSc-ILD samples, possibly related to the elevated pulmonary pressures of these patients (mean pulmonary artery pressure 39.75 mmHg vs 20.25 mmHg in IPF).

### Myeloid Cells

**Figure 2:**
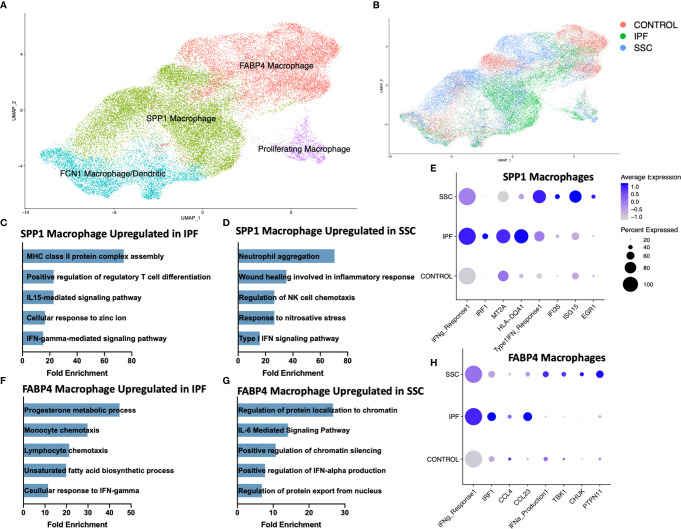
ScRNA-seq analysis of IPF, SSc-ILD, and control myeloid populations. **(A)** UMAP plot of macrophage and dendritic cell populations, identified by cell type. **(B)** UMAP plot of macrophage and dendritic cell populations, identified by disease status. **(C)** Pathways upregulated in IPF *SPP1^hi^* macrophages compared to SSc-ILD *SPP1^hi^* macrophages **(D)** Pathways upregulated in SSc-ILD *SPP1^hi^* macrophages compared to IPF *SPP1^hi^* macrophages **(E)** Expression of selected genes and pathways in *SPP1^hi^* macrophages, separated by disease state. Pathway expression depicted as a module score of all genes in the specific GO biological process. **(F)** Pathways upregulated in IPF *FABP4^hi^* macrophages compared to SSc-ILD *FABP4^hi^* macrophages. **(G)** Pathways upregulated in SSc-ILD *FABP4^hi^* macrophages compared to IPF *FABP4^hi^* macrophages. **(H)** Expression of selected genes and pathways in *FABP4^hi^* macrophages, separated by disease state. scRNA-seq, single-cell RNA-sequencing; IPF, idiopathic pulmonary fibrosis; SSc-ILD, systemic sclerosis-associated interstitial lung disease; UMAP, uniform manifold approximation and projection; GO, gene ontology.

Consistent with our previous scRNA-seq analyses, macrophages appeared in three primary phenotypes: *SPP1* ^hi^ macrophages, *FABP4* ^hi^ macrophages, and a population of monocyte-derived macrophages (*FCN1* ^hi^) (**Figure 2A**, **Supplemental Figure 4**) (31, 32). A small population of dendritic cells clustered within the *FCN1* ^hi^ macrophages. While all three subpopulations of macrophages have been identified in the bronchoalveolar lavage (BAL) fluid of the healthy human lung, *FABP4* ^hi^ macrophages comprised the majority of BAL macrophages, and likely most closely approximate the traditionally designated alveolar macrophages (31). Proliferating macrophages were expanded in IPF and SSc-ILD, compared to controls, with *FABP4* ^hi^ the predominant proliferating phenotype in SSc-ILD and controls, and a similar proportion of proliferating *SPP1* ^hi^ and *FABP4* ^hi^ phenotype cells in IPF. (**Supplemental Figure 5A, B**). Differentially expressed genes and their enhanced gene ontology biological processes were examined between IPF and SSc-ILD lungs for each myeloid population, with the most distinct differences noted amongst the *SPP1* ^hi^ and *FABP4* ^hi^ macrophages (**Figures 2C–H**, **Supplemental Figure 6A, B**). Complete differential expression and gene ontology results for all compared cell populations are included in **Supplemental Files 2** and **3**.

Interferon-gamma (IFN-γ) mediated signaling was distinctly upregulated amongst *SPP1* ^hi^ and *FABP4* ^hi^ macrophages in IPF, compared to SSc-ILD (**Figures 2C, F**). This upregulation included expression of multiple IFN-γ primary response genes such as *IRF1, MT2A, GBP2* and numerous HLA class II antigens within the *SPP1* ^hi^ macrophages and *IRF9, IRF1, CCL3, CCL4*, and *CCL23* within the *FABP4* ^hi^ macrophages (**Figures 2E, H**). Interferon-gamma (*IFNG*) was primarily expressed by natural killer cells and to a lesser extent by T lymphocytes, with macrophages its primary target *via* their expression of the heterodimeric receptor comprised of IFNGR1 and IFNGR2 (**Supplemental Figure 7A, B**). Regulation of T cell differentiation, including that of regulatory T cells, IL-15 mediated signaling, and cellular response to zinc and cadmium ion pathways were also upregulated in IPF macrophages compared to SSc-ILD. Cadmium exposure has recently been implicated as potential exogenous risk factor for pulmonary fibrosis *via* its activation of SMAD2/3/4-dependent signaling in the murine lung (43).

Conversely, type I interferon signaling and production was significantly upregulated amongst *SPP1* ^hi^ and *FABP4* ^hi^ macrophages in SSc-ILD (**Figures 2E, H**), compared to IPF, including expression of multiple interferon-induced transmembrane proteins, *IFI35, ISG15*, and *EGR1* within the *SPP1* ^hi^ macrophages and *TBK1, CHUK*, and *PTPN11*, amongst others, within the *FABP4* ^hi^ macrophages. IL-6 mediated signaling, chromatin silencing, and p53 signal transduction were also upregulated in SSc-ILD *FABP4* ^hi^ macrophages compared to IPF.

### Lymphoid Cells

**Figure 3:**
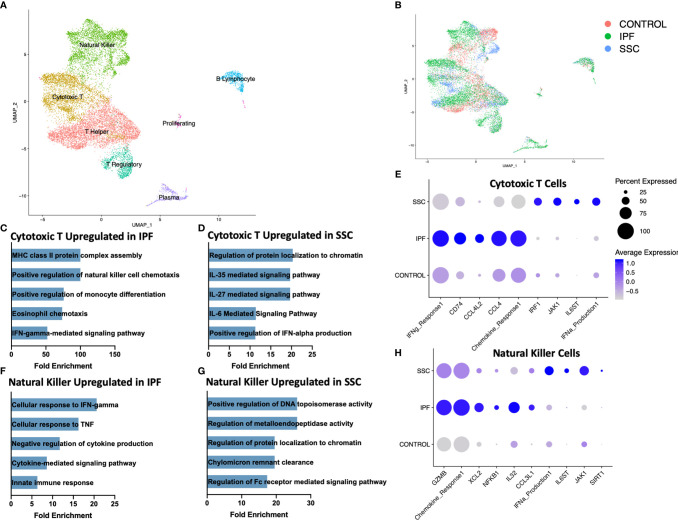
ScRNA-seq analysis of IPF, SSc-ILD, and control lymphoid populations. **(A)** UMAP plot of lymphoid subpopulations, identified by cell type. **(B)** UMAP plot of lymphoid subpopulations, identified by disease status. **(C)** Pathways upregulated in IPF cytotoxic T cells compared to SSc-ILD cytotoxic T cells. If fold enrichment was calculated as >100 it is depicted as 100 for visualization. **(D)** Pathways upregulated in SSc-ILD cytotoxic T cells compared to IPF cytotoxic T cells **(E)** Expression of selected genes and pathways in cytotoxic T cells, separated by disease state. Pathway expression depicted as a module score of all genes in the specific GO biological process. **(F)** Pathways upregulated in IPF NK cells compared to SSc-ILD NK cells **(G)** Pathways upregulated in SSc-ILD NK cells compared to IPF NK cells **(H)** Expression of selected genes and pathways in NK cells, separated by disease state. scRNA-seq, single-cell RNA-sequencing; IPF, idiopathic pulmonary fibrosis; SSc-ILD, systemic sclerosis-associated interstitial lung disease; UMAP, uniform manifold approximation and projection; NK, natural killer.

Reclustering the lymphoid populations, allowed for identification of the three primary T cell populations, as well as improved separation of the natural killer cells, plasma cells, and B lymphocytes (**Figure 3A**, **Supplemental Figure 8**). Natural killer cells and cytotoxic T cells demonstrated the greatest geographic separation in UMAP clustering between IPF and SSc-ILD. We examined differentially expressed genes and their enhanced pathways for each lymphoid population (**Supplemental File 2, 3**, **Supplemental Figure 9**). Amongst the cytotoxic T cells, IFN-γ mediated signaling, regulation of natural killer cell and eosinophil chemotaxis, the ERK1/ERK2 cascade, and responses to IL-1 and tumor necrosis factor (TNF) were distinctly upregulated in IPF compared to SSc-ILD (**Figures 3C, E**, **Supplemental File 3**); identification of these pathways was largely driven by increased expression of numerous major histocompatibility complex (MHC) class II cell surface receptors and several chemokines in IPF cytotoxic T cells. Conversely, IL-6 family mediated signaling pathways (including IL-27 and the related IL-35), as well as positive regulation of type I interferon production were upregulated in SSc-ILD cytotoxic T cells compared to IPF (**Figure 3D**). The proportion of natural killer cells trended toward a graded decline in fibrotic lower lobes compared to fibrotic upper lobes and controls, with a significant decline between controls and SSc-ILD lower lobes (p=0.0379). Cellular response pathways to IFN-γ and TNF, and chemokine response were upregulated in IPF compared to SSc-ILD natural killer cells (**Figures 3F, H**); whereas regulation of metalloendopeptidase activity, and positive regulation of type I interferon production were upregulated in SSc-ILD compared to IPF natural killer cells (**Figures 3G, H**, **Supplemental File 3**).

**Figure 4:**
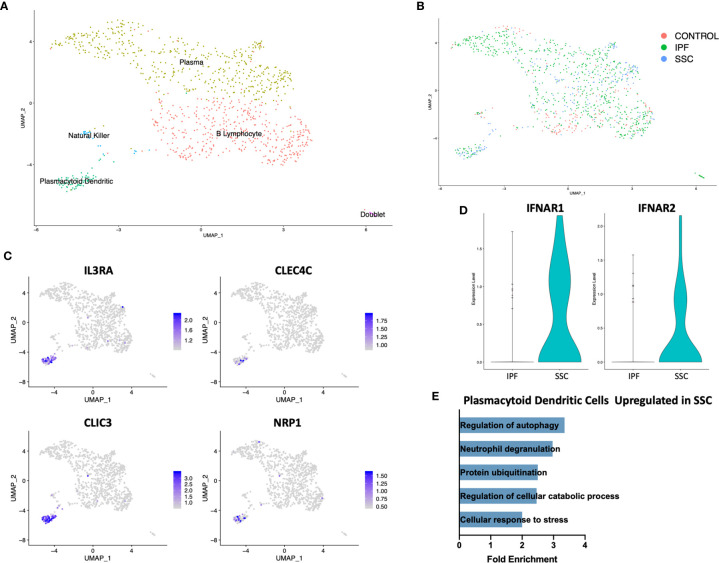
ScRNA-seq analysis of IPF, SSc-ILD, and control plasmacytoid dendritic cell populations. **(A)** UMAP plot of the reclustered original B lymphocyte and plasma cell cluster, identified by cell type. **(B)** UMAP plot of the reclustered original B lymphocyte and plasma cell cluster, identified by disease status. **(C)** Expression of *IL3RA, CLEC4C, CLIC3*, and *NRP1*, identifying pDCs. **(D)** Expression of *IFNAR1*and *IFNAR2* in IPF versus SSc-ILD pDCs. **(E)** Pathways upregulated in SSc-ILD pDCs compared to IPF pDCs. scRNA-seq, single-cell RNA-sequencing; IPF, idiopathic pulmonary fibrosis; SSc-ILD, systemic sclerosis-associated interstitial lung disease; UMAP, uniform manifold approximation and projection; pDCs, plasmacytoid dendritic cells.

On separately reclustering the original B Lymphocyte/plasma cell cluster only, we identified a small population of plasmacytoid dendritic cells (pDC) expressing *IL3RA* (CD123), *CLEC4C, CLIC3*, and *NRP1* (**Figures 4A, C**). This population originated only from IPF and SSc-ILD lungs, with no pDCs identified from control lungs (**Figure 4B**). Multiple cellular stress related pathways including the regulation of autophagy, protein ubiquitination, and response to cellular stress were up-regulated in SSc-ILD pDCs compared to IPF (**Figure 4E**, **Supplemental Figure 10A**), possibly indicating an activated aberrant phenotype amongst the SSc-ILD pDCs. Type 1 interferon receptor expression (*IFNAR1, IFNAR2*) was also increased in SSc-ILD pDCs compared to IPF (**Figure 4D**). We were unable to detect expression of type I IFNs by these cells.

### Epithelial Cells

**Figure 5:**
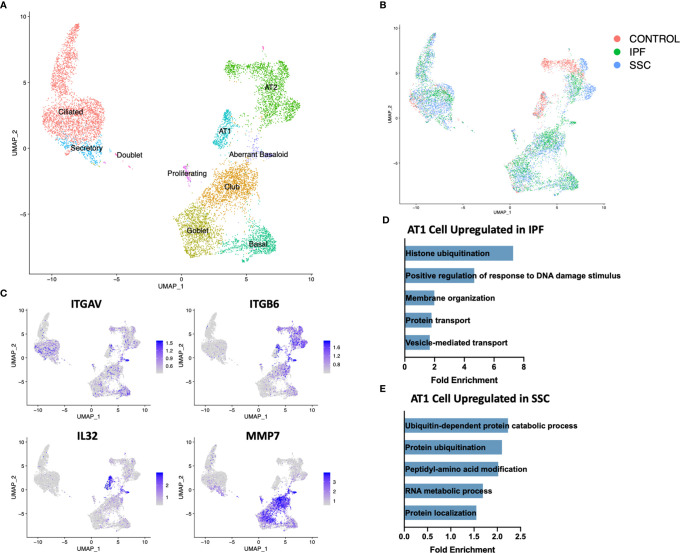
ScRNA-seq analysis of IPF, SSc-ILD, and control epithelial cell populations. **(A)** UMAP plot of epithelial cell populations, identified by cell type **(B)** UMAP plot of epithelial cell populations, identified by disease status. **(C)** Expression of *ITGAV, ITGB6, IL32*, and *MMP7*, highlighting the aberrant basaloid population. **(D)** Pathways upregulated in IPF AT1 cell gene signature compared to SSc-ILD AT1 cell gene signature. **(E)** Pathways upregulated in SSc-ILD AT1 cell gene signature compared to IPF AT1 cell gene signature. scRNA-seq, single-cell RNA-sequencing; IPF, idiopathic pulmonary fibrosis; SSc-ILD, systemic sclerosis-associated interstitial lung disease; UMAP, uniform manifold approximation and projection; AT1, alveolar type 1.

Although genetic evidence has implicated a more central role for epithelial cells in IPF than SSc-ILD, the dramatic loss of alveolar type 1 (AT1) cells and increase in cells originating from the bronchial epithelium was consistent in both diseases (**Figure 1C**, **5B**). Sample digestion and processing may differentially impact the survival of particular cell populations, however the robust presence of AT1 cells within the control samples suggests there is a true loss within the diseased lungs. A population of secretory cells present in disease and controls expressed transcripts associated with both goblet and ciliated cells, including airway mucin *MUC5B*, goblet cell marker *SCGB1A1* and ciliated markers *FOXJ1* and *RSPH1* (**Figure 5A**, **Supplemental Figure 11**).

**Figure 6:**
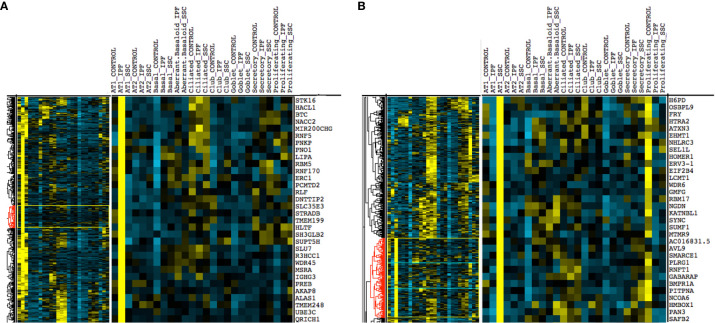
Hierarchical clustering on gene average expression levels of epithelial cell subtypes divided by disease status, demonstrating distinct gene signatures of IPF and SSc-ILD AT1 cells. Yellow indicates positive and blue indicates negative scaled expression. **(A)** Partial gene signature of IPF AT1 cells **(B)** Partial gene signature of SSc-ILD AT1 cells. IPF, idiopathic pulmonary fibrosis; SSc-ILD, systemic sclerosis-associated interstitial lung disease; AT1, alveolar type 1; AT2, alveolar type 2.

Upon hierarchical clustering of the epithelial cells by the average expression of genes within control, IPF, and SSc-ILD samples for each cell type, AT1 cells exhibited the most distinct expression patterns between IPF and SSc-ILD (**Figures 6A, B**). Analyzing the gene signature best distinguishing IPF from SSc-ILD AT1 cells, pathways involved in stress DNA damage response were upregulated in IPF compared to SSc-ILD (**Figures 5D, E**, **Supplemental File 3**), supporting the response to AT1 cellular injury. Multiple E3 ubiquitin-protein ligase genes, including *RNF168, TRIM37, RNF40*, and *HUWE1*, as well as the deubiquitinase *BAP1* were upregulated in IPF AT1 cells, implicating the histone ubiquitination pathway which has been linked to double-stranded DNA break repair as well as transcription repression and activation (44). Other proteins involved in ubiquitination and deubiquitination have been linked to pulmonary fibrosis by both TGF-β dependent and independent mechanisms (45), and further investigation of these AT1 associated ubiquitin proteins may be of particular interest as druggable targets by small molecule inhibitors (46). On the other hand, pathway analysis of SSc AT1 cells indicated altered protein ubiquitination and catabolism, as well as cellular response to oxygen levels, suggesting that cell stress, possibly oxidative stress, leads to death of AT1 cells in SSc-ILD. The distinct transcriptional signatures of AT1 cells between diseases suggests the loss of these cells may results from distinct mechanisms in IPF and SSc-ILD.

AT2 cells, depleted more dramatically in IPF than SSc-ILD, were more transcriptionally similar between IPF and SSc-ILD samples, with few significant pathways identified by genes differentially expressed between IPF and SSc-ILD. The inositol-requiring enzyme 1 (IRE-1) mediated unfolded protein response was upregulated in IPF. IRE1 is an endoplasmic reticulum (ER) stress sensor previously identified to signal through the transcription factor XBP1, as well as the JNK and NF-κB pathways (47). Murine studies suggest a causative role for ER stress in AT2 cell dysfunction, and ER stress is a proposed connection between IPF and multiple fibrosis risk factors including aging, the *MUC5B* risk allele, hypoxia, and infection (47, 48).

Within both the IPF and SSc-ILD samples we identified a small population of the recently described aberrant basaloid cells (or *KRT5^-^/KRT17* ^+^ cells), with no cells sharing this distinct transcriptome amongst the control samples (**Figures 5A, B**) (49, 50). Consistent with recent descriptions in IPF, these cells express traditional basal genes including *KRT17* and *TP63*, but do not express *KRT5, KRT15*, or *SOX2* (**Figure 5C**). In both IPF and SSc-ILD they express elevated *MMP7*, a peripheral blood IPF biomarker, elevated integrin αVβ6, a potent activator of latent TGF-β also implicated in matrix metalloproteinase expression (51), markers of epithelial-mesenchymal transition (such as *COL1A1, CDH2*, and *FN1*), and markers of cellular senescence (including *CDKN2A*(p16), *CDKN1A*(p21), and *GDF15*) (**Supplemental Figure 12**). *IL32* was the top differentially expressed gene amongst the aberrant basaloid cells, compared to the other epithelial cell populations. The presence of aberrant basaloid cells in both IPF and SSc-ILD as well as the severe loss of alveolar type 1 cells, despite purported different drivers, may indicate that both are features of an unsuccessful repair process in advanced lung disease.

### Stromal Populations

**Figure 7:**
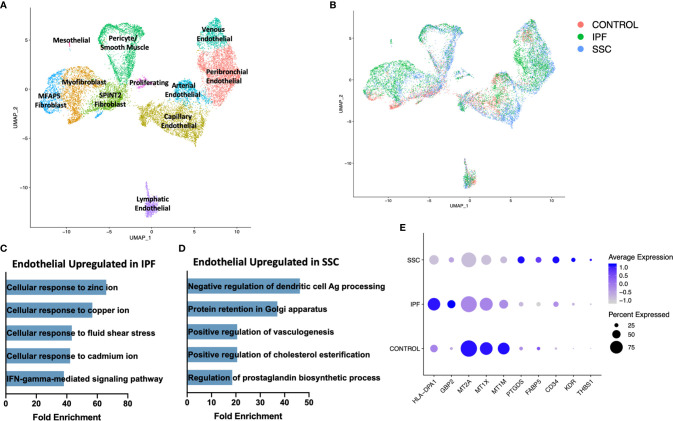
ScRNA-seq analysis of IPF, SSc-ILD, and control stromal cell populations. **(A)** UMAP plot of stromal cell populations, identified by cell type. **(B)** UMAP plot of stromal cell populations, identified by disease status. **(C)** Pathways upregulated in IPF endothelial cells compared to SSc-ILD endothelial cells **(D)** Pathways upregulated in SSc-ILD endothelial cells compared to IPF endothelial cells **(E)** Expression of selected genes in all non-lymphatic endothelial cells, separated by disease state. scRNA-seq, single-cell RNA-sequencing; IPF, idiopathic pulmonary fibrosis; SSc-ILD, systemic sclerosis-associated interstitial lung disease; UMAP, uniform manifold approximation and projection.

On reclustering the stromal populations, three major subpopulations of fibroblasts, pericytes, smooth muscle cells, four major subpopulations of vascular endothelial cells, lymphatic endothelial cells, and mesothelial cells were identified (**Figure 7A**, **Supplemental Figure 13**). A separate cluster of proliferating cells contained myofibroblasts and mixed endothelial cells nearly exclusively originating from IPF and SSc-ILD samples (**Figure 7B**). The major fibroblast subpopulations (myofibroblast, *MFAP5* ^hi^, and *SPINT2* ^hi^) as well as the presence of a small control only *WIF1* ^hi^ population clustering within the myofibroblasts, mirrored those defined in our previous analysis of fibroblasts in SSc-ILD and control lungs (32), even with the addition of cells from the eight IPF samples. The control cells falling within the myofibroblast cluster overall do not express the myofibroblast transcriptome of increased *COL1A1, COL1A2, CTHRC1*, and *POSTN* and instead are more transcriptionally similar to the *SPINT2* ^hi^ fibroblasts.

In comparing all IPF endothelial cells to all SSc-ILD endothelial cells, multiple pathways for cellular responses to metallic ions were distinctly upregulated in IPF (**Figure 7C**, **Supplemental Figure 10B**), driven by the increased expression of multiple metallothionine genes including *MT1A, MT1X, MT1E*, and *MT1M* in IPF endothelial cells, although all were expressed at even higher levels in control cells (**Figure 7E**). Vasculogenesis, prostaglandin biosynthesis, and platelet derived growth factor receptor-signaling were all increased in SSc-ILD compared to IPF (**Figure 7D**, **Supplemental File 3**), reflecting a more active expansion of the endothelium in SSc-ILD. Vasculopathy is understood to play a more central role in the pathogenesis of SSc (12). Thus, these changes may represent important drivers of SSc-PAH associated with SSc-ILD, but also indicate an altered endothelial cell transcriptome that may also play a central role in SSc-ILD pathogenesis. However, since the samples are not matched for physiologic parameters, the observed changes, as well as those of each endothelial subpopulation, (**Supplemental Figures 14**, **15**) cannot be definitively attributed to disease-specific differences.

## Discussion

As the progression of disease can be variable in both IPF and SSc-ILD, our analysis focuses on patients with severe disease resulting in respiratory failure by utilizing only explanted tissues from the time of lung transplant. Although the majority (7 of 8) of our SSc-ILD samples showed UIP on histopathology, we identified significant systematic differences compared to IPF, supporting the notion that many distinct disease mechanisms remain despite the shared histopathologic pattern. Thus, all UIP is not the same at the molecular level, and there is potential loss of scientific knowledge and treatment opportunities in the inclination to think of it as such. The similarities and differences identified between diseases could lead to additional hypotheses regarding treatments that will have a benefit in both diseases versus only IPF or SSc-ILD. We believe that even greater differences could be present in a comparison of early stage disease tissue, however due to the associated risks, patients with SSc-ILD rarely undergo surgical lung biopsy, resulting in no early disease tissue available for research studies.

IPF and SSc-ILD myeloid and lymphoid cells exhibited disparate patterns of interferon signaling, with IFN-γ signaling amplified in IPF *SPP1^hi^* and *FABP4^hi^* macrophages and cytotoxic T and natural killer cells, while Type 1 IFN signaling was amplified in the analogous SSc-ILD populations. In up to 50% of patients with SSc, even early in disease, a type I IFN signature is identifiable in the peripheral blood and correlates with other mediators of fibrosis and inflammation (52, 53). Polymorphisms in several interferon regulatory factors (IRFs) including *IRF4*, *IRF5*, *IRF7*, and *IRF8* have been associated with the development of SSc (24, 54). Type I IFNs in SSc have multiple supported effects including the activation of monocytes, the differentiation and activation of T lymphocytes, B lymphocytes, and dendritic cells, stimulation of the expression of Toll-like receptors by dendritic cells, and increasing the expression of fibrotic effectors such as *CTGF* and *ACTA* in endothelial cells and fibroblasts, amongst others (53). Though the UIP histopathology identified in most of our SSc-ILD samples is often thought of as correlating with less “active inflammation”, and thus being less likely to respond to immunomodulatory therapies, our finding of upregulation of type I IFN in SSc-ILD myeloid and lymphoid populations suggests that type I IFN signaling and inflammation remain an active mechanism in advanced SSc-ILD.

The realm of interferon has previously been considered for therapeutics in IPF, however with a focus on delivering IFN-γ. The 2009 INSPIRE trial evaluated the use of subcutaneous IFN-γ1b in IPF, but was terminated early due to a lack of benefit at interim analysis (55). Inhalational delivery of IFN-γ in IPF has been assessed for safety, and is now being evaluated for a larger trial, with the goal of stimulating macrophages and inhibiting fibrosis *via* this cytokine (56, 57). Albiet aerosolized delivery will likely avoid many of the systemic side effects of parenteral IFN-γ, our findings of upregulation of IFN-γ signaling in the macrophages and selected lymphoid populations of advanced IPF warrant caution in further augmenting this pathway. Although the timing of upregulation of type I and II IFNs during ILD, as well as their role(s) as primary versus secondary drivers of disease is not precisely known, and may vary between IPF and SSc-ILD, ultimately the upregulation of both IFN-γ and Type I IFN signaling may be detrimental in human lung fibrosis.

Plasmacytoid dendritic cells provide a connection between innate and adaptive immunity, and have been identified as producing type 1 interferons during viral infection, and fostering autoimmunity, as well as tolerogenic responses (58). Compared to healthy controls, pDCs are depleted in the blood of patients with SSc, likely secondary to their accumulation within target organs. The frequency of pDCs in BAL fluid correlates with severity of fibrosis on chest CT in SSc-ILD patients (59), while in SSc skin lesions, pDCs accumulate in perivascular regions and produce increased IFN-α and chemokine ligand 4 (CXCL4) *via* TLR8 and phosphoinositide 3-kinase-δ signaling (60, 61). In the murine bleomycin skin and lung fibrosis models, reduction of pDCs results in decreased inflammation and fibrosis, indicating their potential as a therapeutic target in fibrosis (59). Murine studies and the accumulation of conventional dendritic cells (cDCs) in the BAL fluid of patients with IPF have implicated cDC dysfunction in the pathogenesis of IPF, however the role of pDCs in IPF is much less defined (62, 63). In our analysis, although pDCs were recovered from the lung at similar frequencies for both IPF and SSc-ILD, the SSc-ILD pDCs were more transcriptionally active, expressed greater type 1 interferon receptor, and upregulation of multiple cellular stress response pathways. Whether these differences reflect an activated aberrant phenotype of the SSc-ILD pDCs or a failure to act (i.e. senescent-like phenotype) by the IPF pDCs is unclear. Further studies to define the precise function of pDCs and their crosstalk with other cell types within the human fibrotic lung are necessary to discern if these cells are a potential therapeutic target in disease.

Our analysis is the first description of aberrant basaloid cells within the SSc-ILD lung. Although our study includes samples previously analyzed in prior publications by our group, these unique cells were not apparent at that time due to their low cell numbers in analyses with fewer samples. These transcriptionally discrete cells were recently first identified by Adams et al. in a large scRNA-seq analysis of IPF and chronic obstructive pulmonary disease (COPD) lungs (50), as well as by Habermann et al. in a separate large scRNA-seq analysis of pulmonary fibrosis lungs (referred to as *KRT5^-^/KRT17^+^* cells by the latter group) (49). The aberrant basaloid cells express some typical basal cell markers including *TP63* and *KRT17*, but lack others such as *KRT5* and *KRT15*, and also express multiple markers of cellular senescence and epithelial-mesenchymal transition, the highest expression of the IPF biomarker *MMP7*, and elevated expression of the TGF-β activator integrin αVβ6. The lack of aberrant basaloid cells from all control samples and their presence within all IPF and SSc-ILD samples suggests these are a consistent feature of advanced fibrotic disease. Histopathologic examination has suggested these cells localize to the areas of fibroblastic foci in IPF and may activate TGF-β locally *via* their integrin expression (49, 50), now a possible feature in SSc-ILD as well. These cells may exhibit a unique link in the epithelial-mesenchymal transition during which evolving cells lose epithelial characteristics and develop mesenchymal polarity as part of a transition from inflammation to fibrosis (64). The relative proportion of these cells in early versus late disease of both IPF and SSc-ILD is currently unknown, but could help clarify if these cells support ongoing damage and thus are a potential target for therapeutics in both diseases.

Akin to what other scRNA-seq analyses have observed in IPF (50, 65), our analysis identified a dramatic loss of alveolar type I cells in both IPF and SSc-ILD lungs, most significantly in the lower lobes. Despite a similar shift in population prevalence, amongst the epithelial populations the AT1 cells exhibited the most distinct transcriptional signatures between diseases, with upregulation of multiple cellular stress and DNA damage response pathways in IPF compared to SSc-ILD. Current models of IPF pathogenesis suggest repetitive epithelial cell microinjuries induce a dysregulated repair response, prompting a pathological fibrotic response, with the (potentially) intermediary roles for myeloid and lymphoid cells less clear (9). Preclinical models support that epithelial cell injury from environmental exposures may trigger fibrosis, with genetic predisposition and aging creating a more susceptible host to such dysregulated repair (66). Aspiration related to gastroesophageal reflux and esophageal dysmotility is one potential shared injurious mechanism to AT1 cells in IPF and SSc-ILD, although not present in all patients with either disease. While multiple sources of environmental injuries to AT1 cells may exist in SSc-ILD, the downregulation of damage response pathways relative to IPF suggests that their loss may be a secondary effect, rather than an initiating mechanism in SSc-ILD, or occurring due to a very different mechanism, such as injury from immune and/or secondary to endothelial cell dysfunction.

Our study was limited by its small sample size and exclusive use of advanced disease tissue. As patients with IPF and SSc-ILD now rarely undergo surgical lung biopsy, there was no early-stage disease tissue available for analysis. Pulmonary fibrosis, in particular IPF, often develops along an apicobasal gradient, thus we instead included samples from both the upper and lower lobes to capture a greater spectrum of disease. The graded shifts in cell populations most pronounced in the lower lobes, such as the loss of alveolar type 1 and natural killer cells, suggest that this method may enable the identification of progressive changes in the disease course for at least some samples. All IPF and SSc-ILD samples are from patients with end-stage disease receiving care at a tertiary medical center and may not reflect the comprehensive IPF and SSc-ILD population. Due to the limited number of samples, we were unable to perform matching for age, sex, or the degree of pulmonary hypertension. Specifically, the sizeable difference in mean pulmonary artery pressure between the IPF and SSc-ILD patients (20.25 vs 39.75 mmHg) precluded us from determining if differences identified in the endothelial cells, smooth muscle cells, and pericytes are truly disease-specific versus secondary effects of increased pulmonary vascular pressures. To limit confounding from differences in experimental technique, we have chosen to limit our analysis to samples processes with the same digestion protocol and reagent chemistry. Due to the limited number of alveolar type 1 and plasmacytoid dendritic cells recovered from IPF and SSc-ILD samples, analysis of differentially expressed genes may have a higher false positive rate in these populations.

In summary, our analysis utilizes the enhanced capabilities of scRNA-seq to discriminate disease-specific alterations in explanted SSc-ILD and IPF lungs. Macrophages exhibit distinct interferon signatures in IPF versus SSc-ILD, with cytotoxic T cell and natural killer cells expressing similar patterns. The striking loss of alveolar type 1 cells in both diseases likely results from distinct mechanisms, with the presence of aberrant basaloid cells transcriptionally similar to those in IPF now confirmed in SSc-ILD as well. Focusing on shared and unique mechanisms of pathology in IPF and SSc-ILD provides improved insight to identifying treatments that may benefit both diseases. Improved phenotyping of patients with IPF and SSc-ILD to indicate those at highest risk for progressive disease, as well as those with a predominance of specific pathologic mechanisms (such as myofibroblast proliferation or Type 1 IFN signaling) is needed to better guide treatment choices throughout the disease course. Expanding existing lung scRNA-seq datasets and linking these to peripheral blood biomarkers may be a unique opportunity to link pathologic alterations in the tissue to clinically accessible biomarkers, allowing such improved phenotyping.

## Data Availability Statement

The datasets presented in this study can be found in online repositories. The names of the repository/repositories and accession number(s) can be found below: https://www.ncbi.nlm.nih.gov/geo, GSE 128169, GSE 128033, and GSE 156310.

## Author Contributions

EV: design of study, data generation, data analysis, writing of manuscript. TT, AP, JS, HB, MR: data generation and analysis. RL: design of study, interpretation of data and editing manuscript. All authors contributed to the article and approved the submitted version.

## Funding

Research reported in this publication was supported by the National Institutes of Health NIAMS 2P50AR060780 (RL) and NHLBI 2T32HL007563-31 (EV). The content is solely the responsibility of the authors and does not necessarily represent the official views of the National Institutes of Health.

## Conflict of Interest

RL reports grants from Bristol Myers Squibb, Corbus, Formation, Elpidera, Regeneron, Pfizer, and Kiniksa outside the submitted work; personal fees from Bristol Myers Squibb, Formation, Sanofi, Biocon, Boehringer Mannheim, Merck, and Genentech/Roche outside the submitted work.

The remaining authors declare that the research was conducted in the absence of any commercial or financial relationships that could be construed as a potential conflict of interest.
