# Supplementary material for: Disparate Interferon Signaling and Shared Aberrant Basaloid Cells in Single-Cell Profiling of Idiopathic Pulmonary Fibrosis and Systemic Sclerosis-Associated Interstitial Lung Disease
Source: Front Immunol. 2021 Mar 30;12:595811. doi: 10.3389/fimmu.2021.595811 (PMC8042271; doi:10.3389/fimmu.2021.595811)
Supplement: Supplementary file 1 [file DataSheet_1.docx]

Supplemental Methods

**Subjects and tissue preparation**

The University of Pittsburgh Institutional Review Board approved procedures involving human samples. All samples were obtained at the University of Pittsburgh Medical Center. We performed sampling of the explanted lung(s) in the operating room for patients with IPF and SSc-ILD at the time of lung transplantation. A designated researcher from our team received the lung explant in the operating room immediately after transfer of the lung away from the surgical field. Without any break in sterility, we resected 3-4 pieces of subpleural lung tissue from a lower lobe and upper lobe (either left or right, whichever was removed first during surgery) from all explants included in the study. The same researcher performed the operating room collection for all samples included in this study. The remaining lung explant(s) were then available to the treating physicians for standard pathology studies. Tissue sections were placed in Perfadex and arrived for immediate processing in our laboratory (PI- Dr. Robert Lafyatis) within twenty minutes of removal from the patient. All samples were processed (from tissue digestion through barcoded cDNA creation) in the same laboratory immediately after tissue collection. Library preparation was performed within 7 days of sample acquisition.

Sections were allocated for formalin fixation, preservation in RNA Later, and immediate digestion for scRNA-seq. To prepare the single-cell suspension, tissue 1-5cm from the pleural membrane was diced then enzymatically digested in DMEM (Thermo Fisher Scientific) containing 0.7 mg/mL collagenase A (Roche) and 30 ug/mL DNAase I (Roche) for one hour while undergoing further mechanical dispersion with the gentleMACS OctoDissociator (Miltenyi Biotec). The resulting cell suspension was washed with PBS, filtered twice through a 70-micron cell strainer, and underwent RBC lysis. Cells were then resuspended in PBS containing 0.04% BSA. ScRNA-seq library preparation was performed using the 10X Genomics Chromium System and its associated V2 chemistry reagents per the manufacturer’s protocol.

Control lung tissue was obtained from explanted lungs procured from organ donation candidates that were deemed unsuitable for lung transplantation. These lungs were obtained through the Pennsylvania Center for Organ Recovery and Education (CORE). All patients met brain death criteria, and these samples were anonymized by CORE investigators. For control lungs, we also obtained tissue samples in the operating room under sterile conditions as detailed above. We made every effort to sample macroscopically normal appearing lung tissue from the subpleural portions of the upper and lower lobes, in order to match the corresponding regions sampled from the diseased lungs.

**Single-cell RNA library preparation and sequencing**

Using the 10X Genomics Chromium System, cells were mixed with reverse transcription reagents, loaded into a Single-cell A chip, followed by 3’ gel beads and partitioning oil. Cells were separated into oil micro-droplet partitions containing a cell and gel-bead scaffold for an oligonucleotide composed of oligo-DT, 10X and UMI barcodes, and reverse transcription reagents. Reverse Transcription was performed, the emulsion broken, and pooled fractions obtained using a recovery agent. cDNAs were amplified by 11 cycles of PCR (C1000, Bio-Rad), enzymatically sheared and DNA fragment ends were repaired, A-tailed and adaptors ligated. The library was quantified with the KAPA Universal Library Quantification Kit KK4824 (KAPA Biosystems) and evaluated for cDNA length on a bioanalyzer using a High Sensitivity DNA kit (Agilent). ScRNA-seq libraries for the 13 samples in the primary analysis were sequenced on an Illumina NextSeq-500 through the University of Pittsburgh Genomics Core Sequencing Facility. Raw sequencing reads were examined by quality metrics and mapped to human reference genome GRCh38 using the Cell Ranger pipeline (10X Genomics). To ensure PCR amplified transcripts were counted only once, only single UMIs were counted for gene expression level. In this way, cell x UMI count matrices were generated for downstream analysis.

**Combined analysis of multiple samples**

Cells were filtered for greater than 200 genes, less than 50,000 UMIs, and less than 15 percent mitochondrial genes. To minimize batch effects in combining multiple samples for integrated analysis, an individual object was created for each sample then all control, IPF, and SSc-ILD samples were integrated into 3 objects by disease status using Seurat’s IntegrateData function. The control, IPF and SSc-ILD object were then integrated to one final object for the overall clustering. Integration anchors were identified then a joint structure learned for the comprehensive dataset via canonical correlation analysis. Following integration the data was normalized (LogNormalized with a scale factor of 10,000), variable features identified, scaled (with regression of percent mitochondrial genes and number of UMI), and principle component analysis (PCA) run. The dimensionality of the dataset was examined by heatmap, JackStraw, and elbow plots for selection of principal components for clustering analysis. The standard workflow Seurat v3 clustering method was used for clustering, namely a K-nearest neighbor graph was constructed based on Euclidean distance in PCA space with edge weights refined by Jaccard similarity, followed by modularity optimization by the Louvain algorithm to iteratively group cells together.[35] The use of 27 principal components with a resolution of 0.5 produced a maximum modularity of 0.9429 for 26 clusters (Supplemental Figure 2). Including additional principal components did not significantly alter the clustering, and further increasing the resolution resulted in greater separation of clusters by individual sample.

Following visualization with uniform manifold approximation and projection (UMAP),[36] cell populations were classified using multiple gene markers in the transcriptome (Supplemental Table 3), and aggregated into overall cell types for visualization (Figure 1A) and subclustering. Despite initial filtering, two clusters (clusters 7 and 15) contained lower-quality cells from mixed cell types, based on their low-expression of a smaller number of genes and low average UMIs compared to the other clusters. Doublet cells were manually identified as expressing markers of multiple cell types with elevated UMI counts and subsequently removed. Cell cycle phase was predicted using Seurat’s CellCycleScoring function. Regressing out cell phase did not significantly alter the data architecture (Supplemental Figure 16). To better define individual cell types, the myeloid, lymphoid, epithelial, and stromal populations were then separately reclustered in a similar manner. The number of principal components used and resolution for each subclustering was selected to optimize separation of biologically relevant cell types while minimizing separation by individual sample. When reclustering limited cell types, if a distinct group of lower quality cells was identified (empty droplets, damaged, and dying cells) they were removed from further analysis.

**Supplemental Table 1**

Cell numbers by sample post filtering (number of genes >200, number of UMI <50,000, percent mitochondrial genes <15%), as well as number of cells clustering in the “low-quality” cluster for each sample.

| Sample | Cells post- filtering | Remaining low quality cells post-filtering |
| --- | --- | --- |
| IPF 1 | 4577 | 186 |
| IPF 2 | 5931 | 364 |
| IPF 3 | 2233 | 140 |
| IPF 4 | 3205 | 148 |
| IPF 5 | 4311 | 250 |
| IPF 6 | 3491 | 177 |
| IPF 7 | 4502 | 308 |
| IPF 8 | 3717 | 370 |
| SSC 1 | 4110 | 164 |
| SSC 2 | 3988 | 196 |
| SSC 3 | 3735 | 359 |
| SSC 4 | 3205 | 237 |
| SSC 5 | 5276 | 641 |
| SSC 6 | 4647 | 305 |
| SSC 7 | 3741 | 378 |
| SSC 8 | 3605 | 310 |
| CONTROL 1 | 3435 | 172 |
| CONTROL 2 | 4422 | 341 |
| CONTROL 3 | 3307 | 78 |
| CONTROL 4 | 5983 | 440 |
| CONTROL 5 | 4335 | 211 |

**Supplemental Table 2**

Cell numbers (inclusive of control, IPF, and SSc-ILD cells) by cell subpopulation and number of differentially expressed genes (DEGs), with p-value<0.05 and absolute avglogFC>0.5, included in gene ontology pathway analyses.

| Cell Population | Number of Cells | Number of DEGs upregulated in IPF | Number of DEGs  Upregulated in SSC |
| --- | --- | --- | --- |
| SPP1 Macrophage | 16583 | 214 | 289 |
| FABP4 Macrophage | 13132 | 96 | 743 |
| Monocyte/Dendritic | 6116 | 40 | 179 |
| Proliferating Macrophage | 1309 |  |  |
| Mast | 2074 |  |  |
| Endothelial | 8576 | 74 | 336 |
| Arterial Endothelial | 1098 | 116 | 960 |
| Capillary Endothelial | 3133 | 101 | 477 |
| Peribronchial Endothelial | 3156 | 90 | 213 |
| Venous Endothelial | 1189 | 46 | 797 |
| Lymphatic Endothelial | 847 | 92 | 794 |
| Fibroblast | 4687 |  |  |
| MFAP5 Fibroblast | 1069 |  |  |
| Myofibroblast | 2019 |  |  |
| SPINT2 Fibroblast | 1599 |  |  |
| Smooth Muscle/Pericyte | 2416 |  |  |
| Mesothelial | 57 |  |  |
| Ciliated | 3017 | 1581 | 420 |
| Club | 1656 | 84 | 119 |
| Goblet | 1339 | 161 | 67 |
| Secretory | 365 | 455 | 391 |
| Basal | 885 | 242 | 50 |
| Alveolar Type 2 | 1864 | 135 | 379 |
| Alveolar Type 1 | 462 | 577 | 1030 |
| Aberrant Basaloid | 159 | 352 | 506 |
| T Helper | 4837 | 33 | 87 |
| Cytotoxic T | 3379 | 48 | 387 |
| Natural Killer | 3684 | 31 | 398 |
| T Regulatory | 937 | 58 | 299 |
| B Lymphocyte | 629 | 237 | 202 |
| Plasma | 433 | 359 | 121 |
| Plasmacytoid DC | 67 | 149 | 262 |

**Supplemental Table 3**

Marker genes used for cell population annotation.

| **Cell Type** | **Marker Genes** |
| --- | --- |
| **Myeloid** | AIF1, PTPRC |
| Macrophage | AIF1, FCGR1A, CD163 |
| *SPP1*^hi^ Macrophage | SPP1, MERTK, CCL2 |
| *FABP4*hi Macrophage | FABP4, INHBA |
| Monocyte | FCN1, IL1B, CD14 |
| Dendritic | CD1C, PLD4, CLEC91, FCER1A, |
| Mast | TPSAB1, CPA3, MS4A2, KIT |
| **Lymphoid** | PTPRC, CD3E |
| Cytotoxic T | CD3D, CD8A, CD8B, GZMK |
| Helper T | CD4, COTL1^hi^, CCR7, LEF1 |
| T Regulatory | FOXP3 |
| Natural Killer | NKG7^hi^, KLRF1, FCER1G, TYROBP |
| B Lymphocyte | MS4A1, CD79A, CD19 |
| Plasma | JCHAIN, CD79A, IGLL5 |
| Plasmacytoid Dendritic | CLEC4C, CLIC3, NRP1, IL3RA |
| **Epithelial** | EPCAM |
| Alveolar Type 1 | AGER, PDPN, CLIC5 |
| Alveolar Type 2 | SFTPC, SFTPD, MUC1^hi^ |
| Basal | KRT5, KRT15, KRT17, TP63 |
| Aberrant Basaloid | KRT17, MMP7, COL1A1, FN1 |
| Ciliated | RSPH1, CAPS, FOXJ1 |
| Club | SCGB3A2 |
| Goblet | MUC5B, SCGB1A1, MUC5AC, SPDEF |
| Secretory | SCGB1A1, MUC5B, SPDEF |
| **Mesenchymal** |  |
| Endothelial | VWF, PECAM1, CLDN5 |
| Arterial Endothelial | DKK2, IGFBP3, GJA5, BMX |
| Capillary Endothelial | HPGD, IL7R, CA4, FCN3hi, SEMA3G |
| Peribronchial Endothelial | COL15A1, PLVAP^hi^, MPZL2, POSTN |
| Venous Endothelial | HDAC9, ACKR1^hi^, SELE^hi^, SULT1E1 |
| Lymphatic Endothelial | LYVE1, CCL21, PROX1 |
| Fibroblast | LUM, PDGFRA, COL1A2 |
| Myofibroblast | ACTA2^hi^, POSTN, CTHRC1 |
| *MFAP5*hi Fibroblast | MFAP5, PLA2G2A, CXCL14 |
| *SPINT2*hi Fibroblast | SPINT2, CD14, LMCD1^hi^, AOC3^hi^, FGFR4^hi^ |
| Smooth Muscle | DES, ACTA2^hi^, MYH11 |
| Pericyte | PDGFRB^hi^, RGS5, FAM162B, |
| Mesothelial | UPK3B, WT1, MSLN |
| Proliferating Cells | TOP2A, BIRC5, UBE2C, MKI67 |

**Supplemental Table 4.**

Key for matching sample IDs from manuscript to sample designations in GEO

| Sample ID | GEO Sample |
| --- | --- |
| CONTROL 1 | SC45 |
| CONTROL 2 | SC56 |
| CONTROL 3 | SC59 |
| CONTROL 4 | SC156 |
| CONTROL 5 | SC155 |
| SSC 1 | SC52 |
| SSC 2 | SC51 |
| SSC 3 | SC64 |
| SSC 4 | SC63 |
| SSC 5 | SC109 |
| SSC 6 | SC108 |
| SSC 7 | SC136 |
| SSC 8 | SC135 |
| IPF 1 | SC88 |
| IPF 2 | SC87 |
| IPF 3 | SC94 |
| IPF 4 | SC93 |
| IPF 5 | SC154 |
| IPF 6 | SC153 |
| IPF 7 | SC175 |
| IPF 8 | SC174 |
