# Supplementary material for: Disparate Interferon Signaling and Shared Aberrant Basaloid Cells in Single-Cell Profiling of Idiopathic Pulmonary Fibrosis and Systemic Sclerosis-Associated Interstitial Lung Disease
Source: Front Immunol. 2021 Mar 30;12:595811. doi: 10.3389/fimmu.2021.595811 (PMC8042271; doi:10.3389/fimmu.2021.595811)
Supplement: Supplementary file 2 [file DataSheet_2.pdf]

Supplemental Figure Legends

Supplemental Figure 1. H&E stained histopathology of adjacent lung tissue sections from the explanted lungs, identified by sample id. Scale bar=1mm. IPF, idiopathic pulmonary fibrosis; SSc, systemic sclerosis.

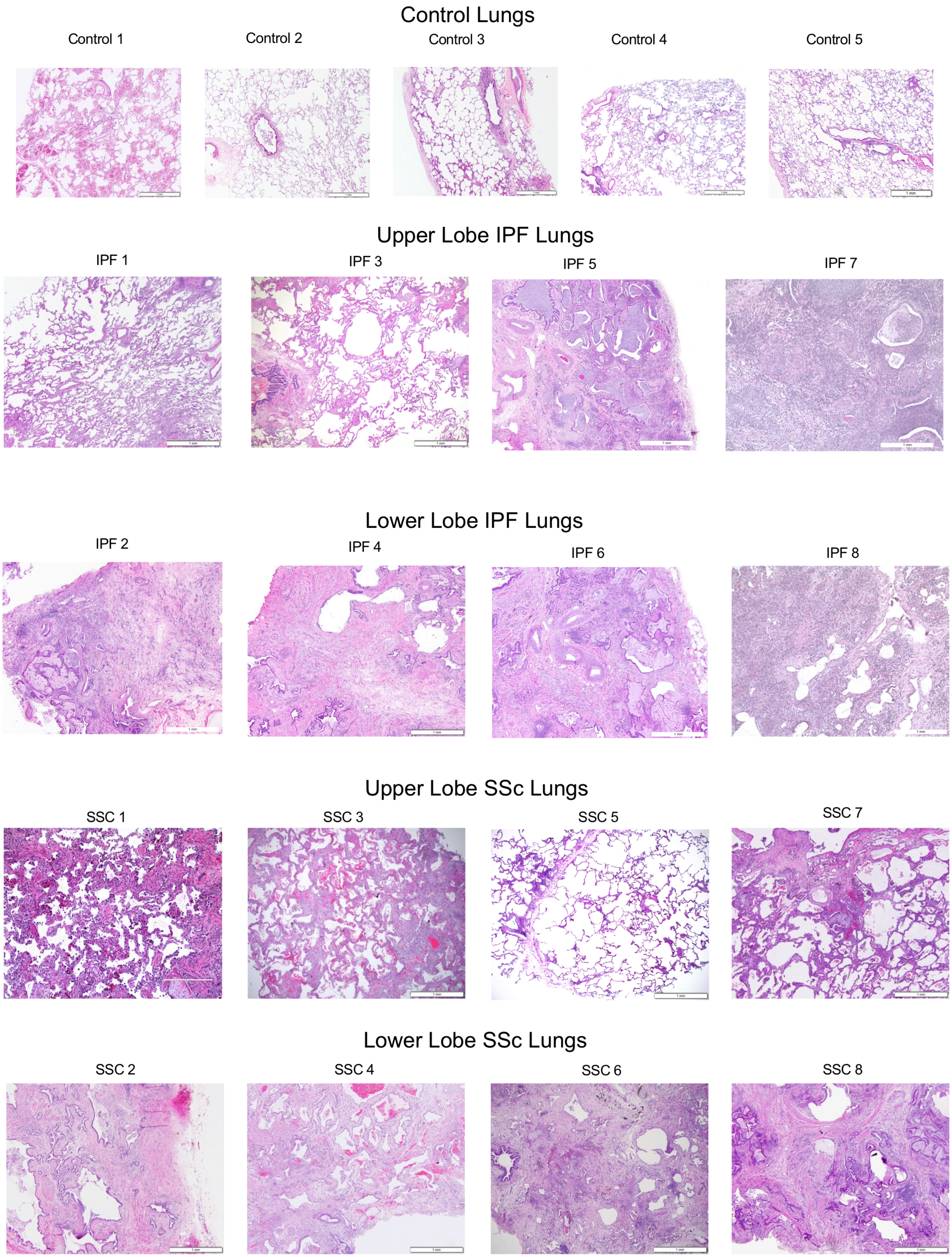
Control Lungs
Control 1
Control 2
Control 3
Control 4
Control 5
Upper Lobe IPF Lungs
IPF 1
IPF 3
IPF 5
IPF 7
Lower Lobe IPF Lungs
IPF 2
IPF 4
IPF 6
IPF 8
Upper Lobe SSc Lungs
SSC 1
SSC 3
SSC 5
SSC 7
Lower Lobe SSc Lungs
SSC 2
SSC 4
SSC 6
SSC 8

Supplemental Figure 2. ScRNA-seq analysis of 8 IPF, 8 SSc-ILD, and 5 organ donor control lung samples. (A) UMAP plot of all 21 integrated samples, with original 26 clusters, prior to aggregation by cell type. (B) UMAP plots of all samples, labeled by individual sample. scRNA-seq, single-cell RNA-sequencing; IPF, idiopathic pulmonary fibrosis; SSc-ILD, systemic sclerosis-associated interstitial lung disease; UMAP, uniform manifold approximation and projection.

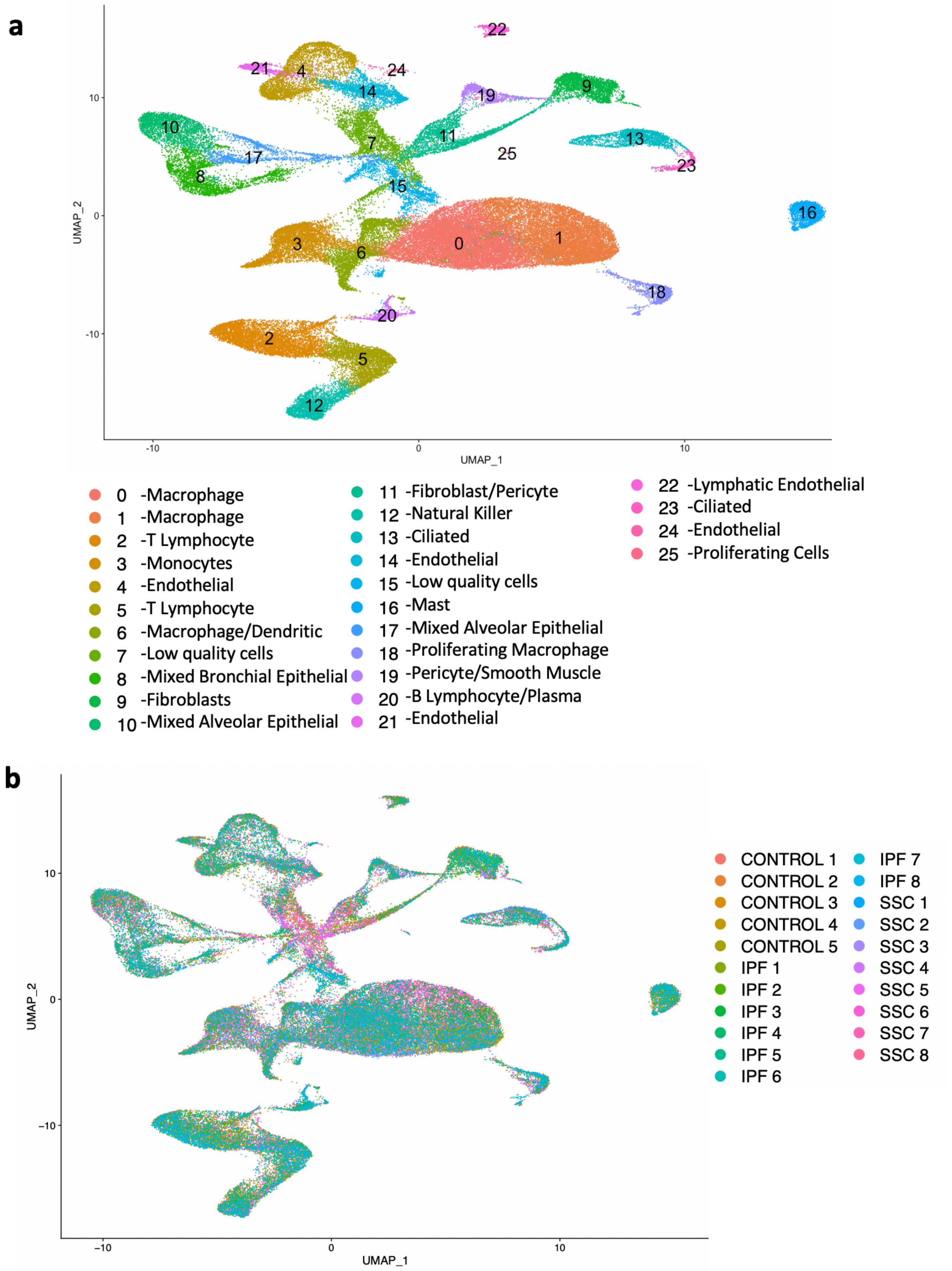
a
UMAP_2
UMAP_1
0 -Macrophage
1 -Macrophage
2 -T Lymphocyte
3 -Monocytes
4 -Endothelial
5 -T Lymphocyte
6 -Macrophage/Dendritic
7 -Low quality cells
8 -Mixed Bronchial Epithelial
9 -Fibroblasts
10 -Mixed Alveolar Epithelial
11 -Fibroblast/Pericyte
12 -Natural Killer
13 -Ciliated
14 -Endothelial
15 -Low quality cells
16 -Mast
17 -Mixed Alveolar Epithelial
18 -Proliferating Macrophage
19 -Pericyte/Smooth Muscle
20 -B Lymphocyte/Plasma
21 -Endothelial
22 -Lymphatic Endothelial
23 -Ciliated
24 -Endothelial
25 -Proliferating Cells
b
UMAP_2
UMAP_1
CONTROL 1
CONTROL 2
CONTROL 3
CONTROL 4
CONTROL 5
IPF 1
IPF 2
IPF 3
IPF 4
IPF 5
IPF 6
IPF 7
IPF 8
SSC 1
SSC 2
SSC 3
SSC 4
SSC 5
SSC 6
SSC 7
SSC 8

Supplemental Figure 3. ScRNA-seq analysis of IPF, SSc-ILD, and control cells with expression of selected cell type markers. *AIF1*- macrophages; *CD3E*-lymphoid cells; *NKG7*- natural killer cells; *MS4A1*- B lymphocytes; *IGLL5*- plasma cells; *EPCAM*-epithelial cells; *CAPS*- ciliated cells; *VWF*-endothelial cells; *CCL21*-lymphatic endothelial cells; *LUM*-fibroblasts; *DES*- smooth muscle cells; *PDGFRB*- pericytes; *KIT*-mast cells; *TOP2A*- proliferating cells; *BIRC5*-proliferating cells. scRNA-seq, single-cell RNA-sequencing; IPF, idiopathic pulmonary fibrosis; SSc-ILD, systemic sclerosis-associated interstitial lung disease.

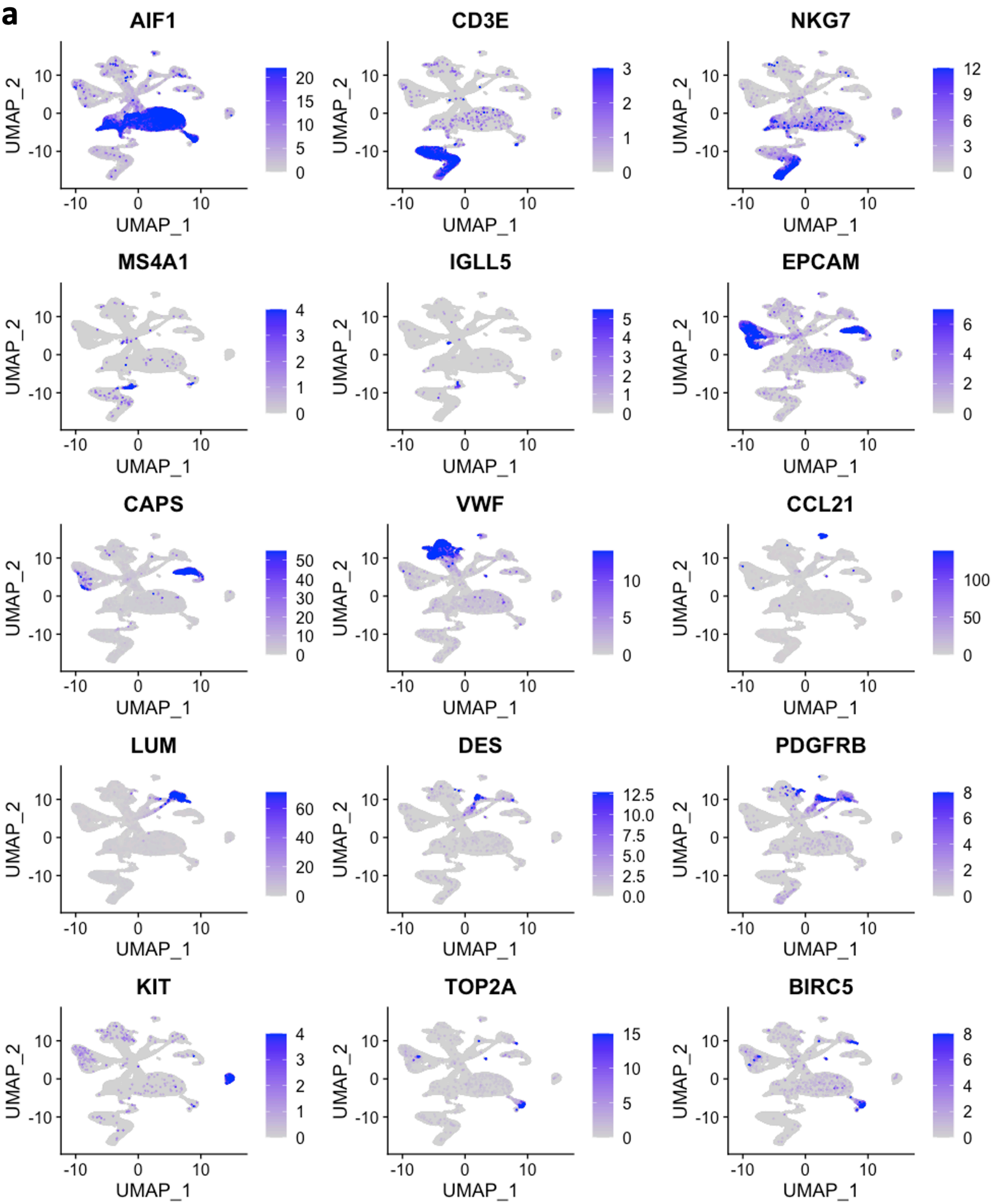
a
AIF1
CD3E
NKG7
MS4A1
IGLL5
EPCAM
CAPS
VWF
CCL21
LUM
DES
PDGFRB
KIT
TOP2A
BIRC5
UMAP_1
UMAP_2

Supplemental Figure 4. ScRNA-seq analysis of IPF, SSc-ILD, and control myeloid populations. Expression of *SPP1*, *MERTK*, and *CCL2* indicate *SPP1*^{hi} macrophages. Expression of *FABP4* and *INHBA* indicate *FABP4*^{hi} macrophages. Expression of *FCN1* and *IL1B* indicate *FCN1*^{hi} macrophages. Expression of *CD1C* indicates dendritic cells. Expression of *BIRC5* and *TOP2A* indicate proliferating macrophages. scRNA-seq, single-cell RNA-sequencing; IPF, idiopathic pulmonary fibrosis; SSc-ILD, systemic sclerosis-associated interstitial lung disease.

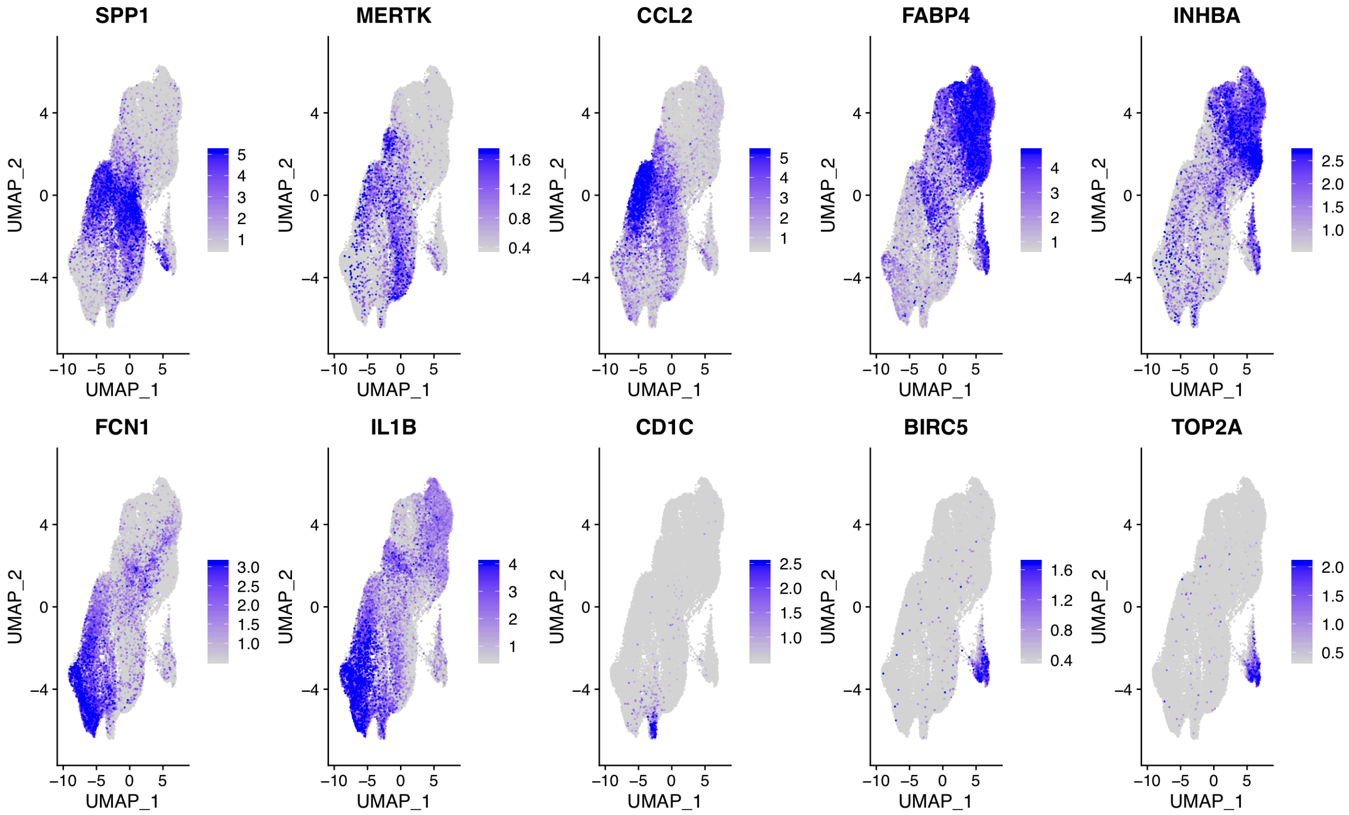
SPP1
MERTK
CCL2
FABP4
INHBA
FCN1
IL1B
CD1C
BIRC5
TOP2A
UMAP_1
UMAP_2

Supplemental Figure 5. ScRNA-seq analysis of IPF, SSc-ILD, and control myeloid populations. (A) UMAP plot of myeloid populations, identified by predicted cell phase. (B) Expression of macrophage subpopulation markers *SPP1*, *FABP4*, and *FCN1* in the proliferating macrophage cluster only, separated by disease status. scRNA-seq, single-cell RNA-sequencing; IPF, idiopathic pulmonary fibrosis; SSc-ILD, systemic sclerosis-associated interstitial lung disease; UMAP, uniform manifold approximation and projection.

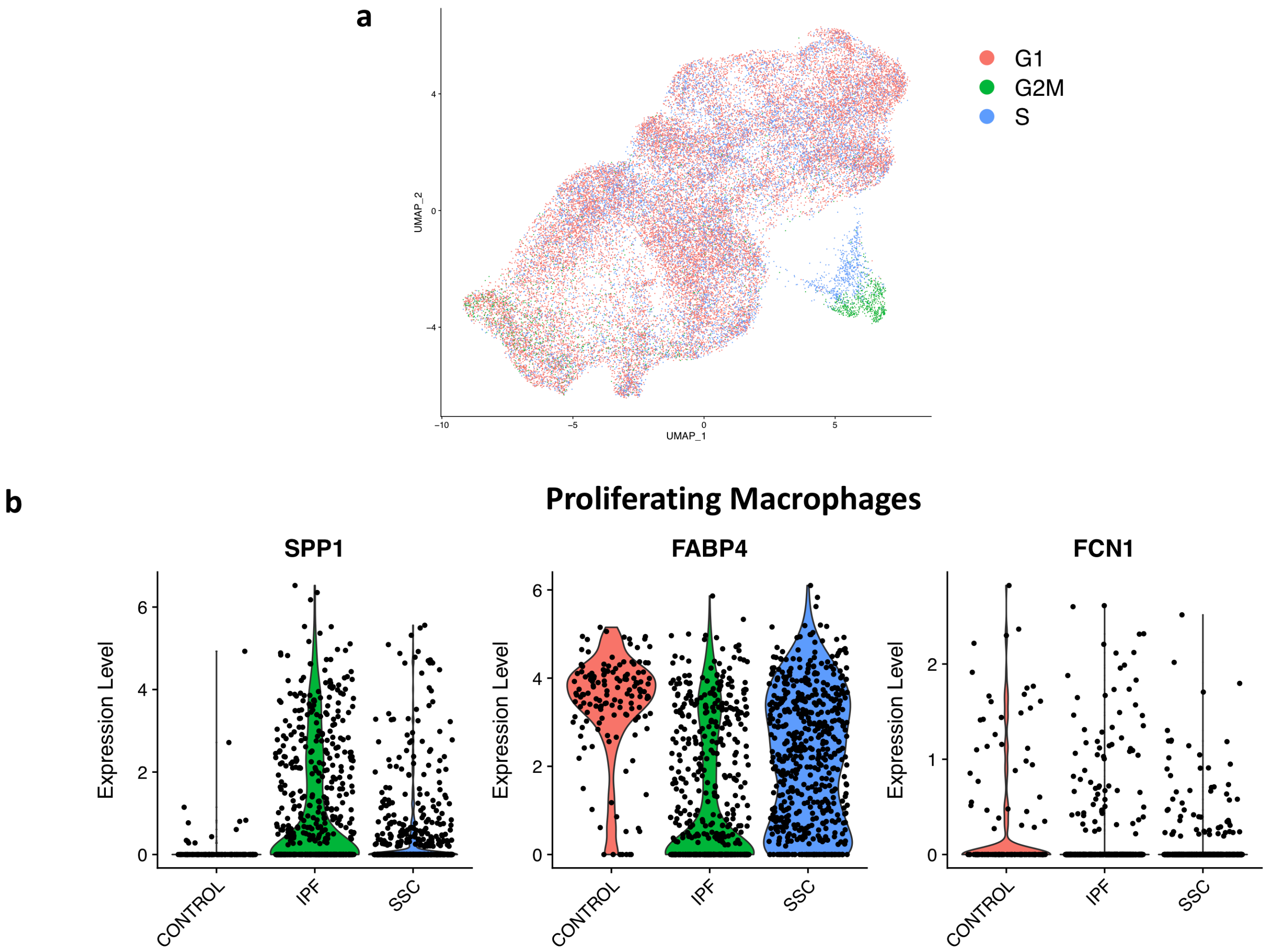
a
G1
G2M
S
UMAP_2
UMAP_1
b
Proliferating Macrophages
SPP1
FABP4
FCN1
Expression Level
CONTROL
IPF
SSC

Supplemental Figure 6. (A) Heat map of *SPP1*^hi macrophages, labeled by disease status, displaying 50 differentially expressed genes for the comparison of IPF and SSc-ILD cells. (B) Heat map of *FABP4*^hi macrophages, labeled by disease status, displaying 50 differentially expressed genes for the comparison of IPF and SSc-ILD cells.

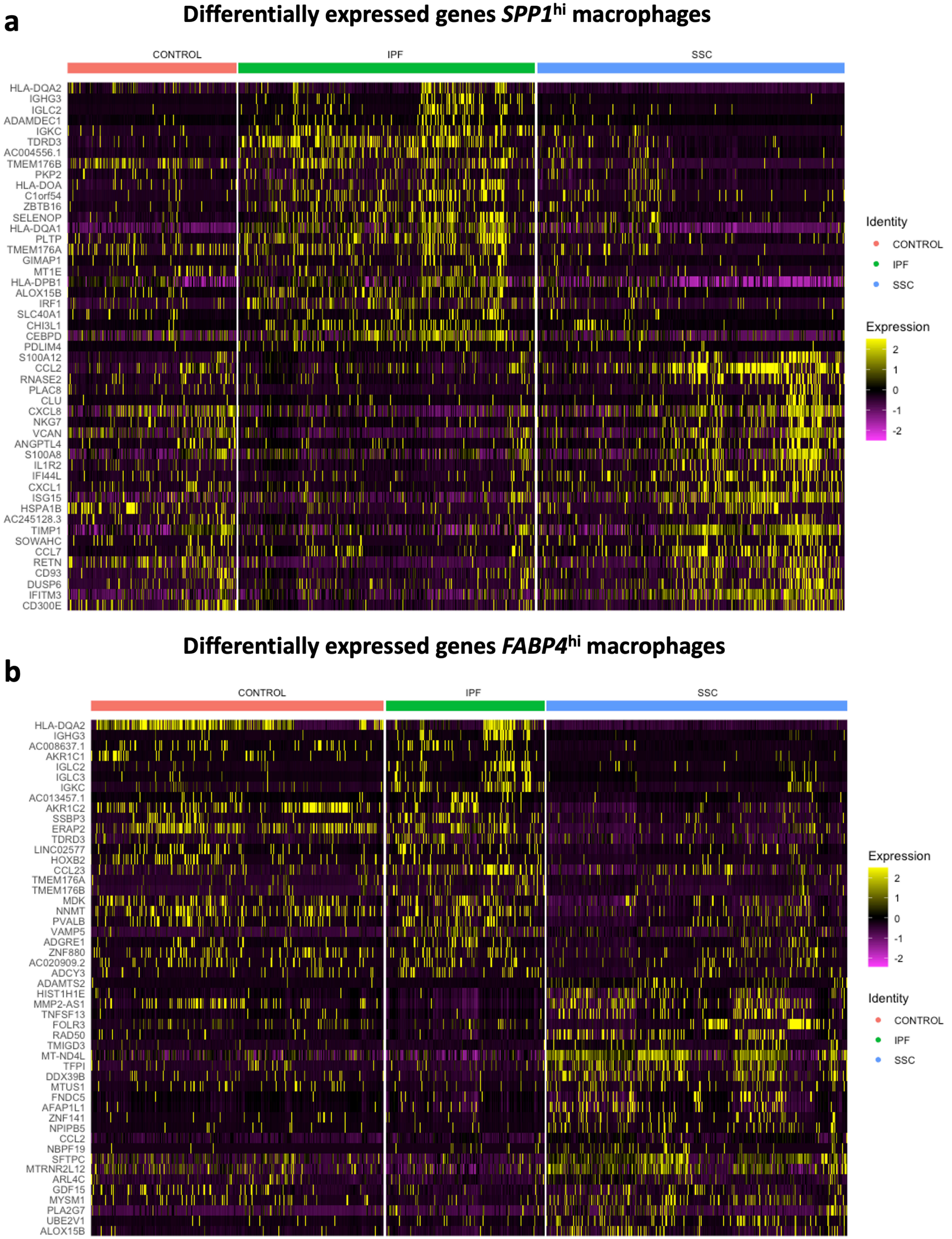
a
Differentially expressed genes SPP1hi macrophages
CONTROL
IPF
SSC
HLA-DQA2
IGHG3
IGLC2
ADAMDEC1
IGKC
TDRD3
AC004556.1
TMEM176B
PKP2
HLA-DOA
C1orf54
ZBTB16
SELENOP
HLA-DQA1
PLTP
TMEM176A
GIMAP1
MT1E
HLA-DPB1
ALOX15B
IRF1
SLC40A1
CHI3L1
CEBPD
PDLIM4
S100A12
CCL2
RNASE2
PLAC8
CLU
CXCL8
NKG7
VCAN
ANGPTL4
S100A8
IL1R2
IFI44L
CXCL1
ISG15
HSPA1B
AC245128.3
TIMP1
SOWAHC
CCL7
RETN
CD93
DUSP6
IFITM3
CD300E
Identity
CONTROL
IPF
SSC
Expression
2
1
0
-1
-2
b
Differentially expressed genes FABP4hi macrophages
CONTROL
IPF
SSC
HLA-DQA2
IGHG3
AC008637.1
AKR1C1
IGLC2
IGLC3
IGKC
AC013457.1
AKR1C2
SSBP3
ERAP2
TDRD3
LINC02577
HOXB2
CCL23
TMEM176A
TMEM176B
MDK
NNMT
PVALB
VAMP5
ADGRE1
ZNF880
AC020909.2
ADCY3
ADAMTS2
HIST1H1E
MMP2-AS1
TNFSF13
FOLR3
RAD50
TMIGD3
MT-ND4L
TFPI
DDX39B
MTUS1
FNDC5
AFAP1L1
ZNF141
NPIPB5
CCL2
NBPF19
SFTPC
MTRNR2L12
ARL4C
GDF15
MYSM1
PLA2G7
UBE2V1
ALOX15B
Expression
2
1
0
-1
-2
Identity
CONTROL
IPF
SSC

Supplemental Figure 7. (A) UMAP plot of all cell type populations with expression of *IFNG* highest in the natural killer cells, and expression of *IFNGR1* and *IFNGR2* highest in the macrophages. (B) UMAP plot of the lymphoid populations indicating expression of *IFNG* is highest in the natural killer cells, and also expressed in the T lymphocytes. UMAP, uniform manifold approximation and projection.

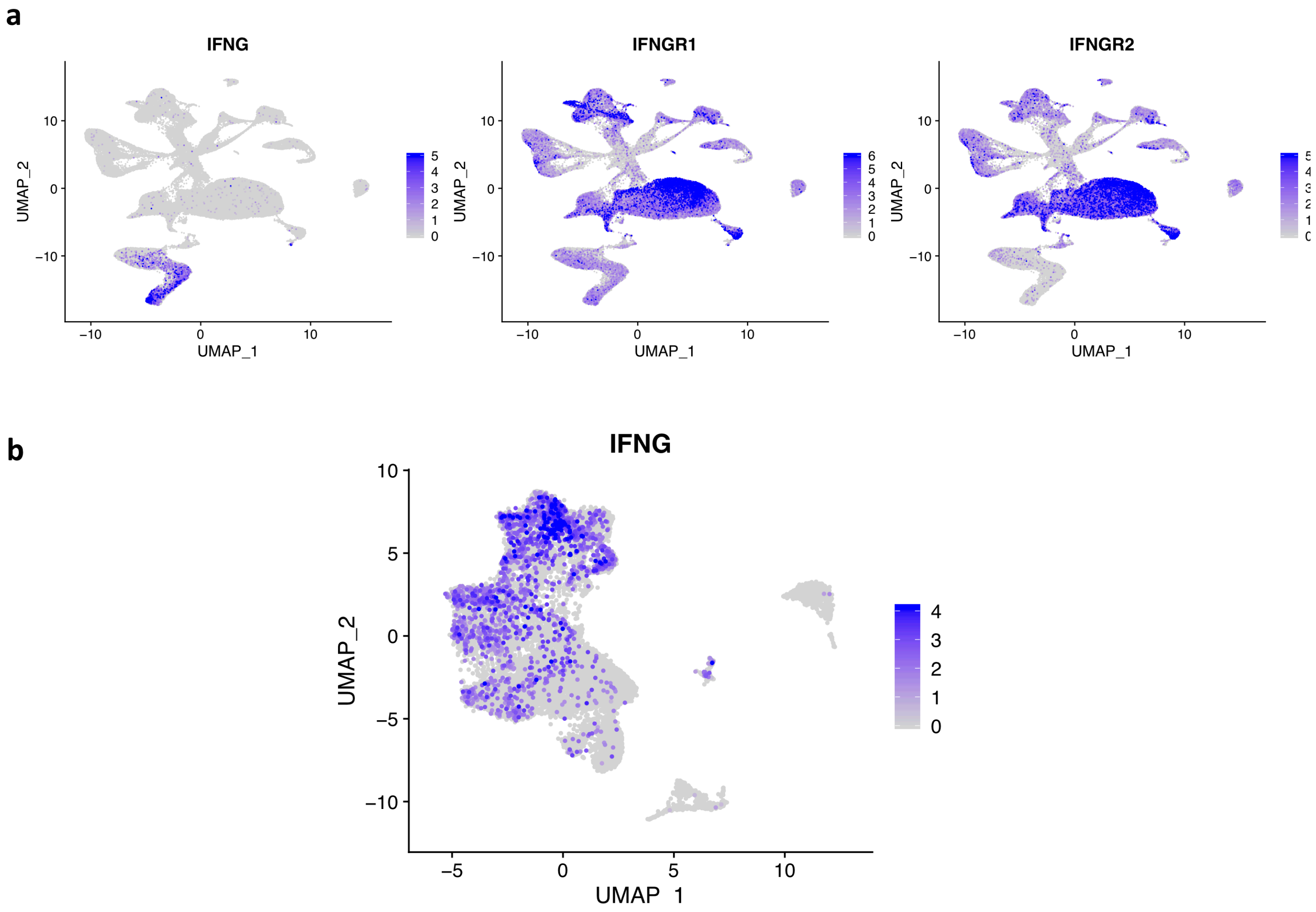

a
IFNG
IFNGR1
IFNGR2
UMAP_1
UMAP_2
b
IFNG
UMAP_2
UMAP 1

Supplemental Figure 8. Violin plots demonstrating expression of selected marker genes for the lymphoid populations clustering.

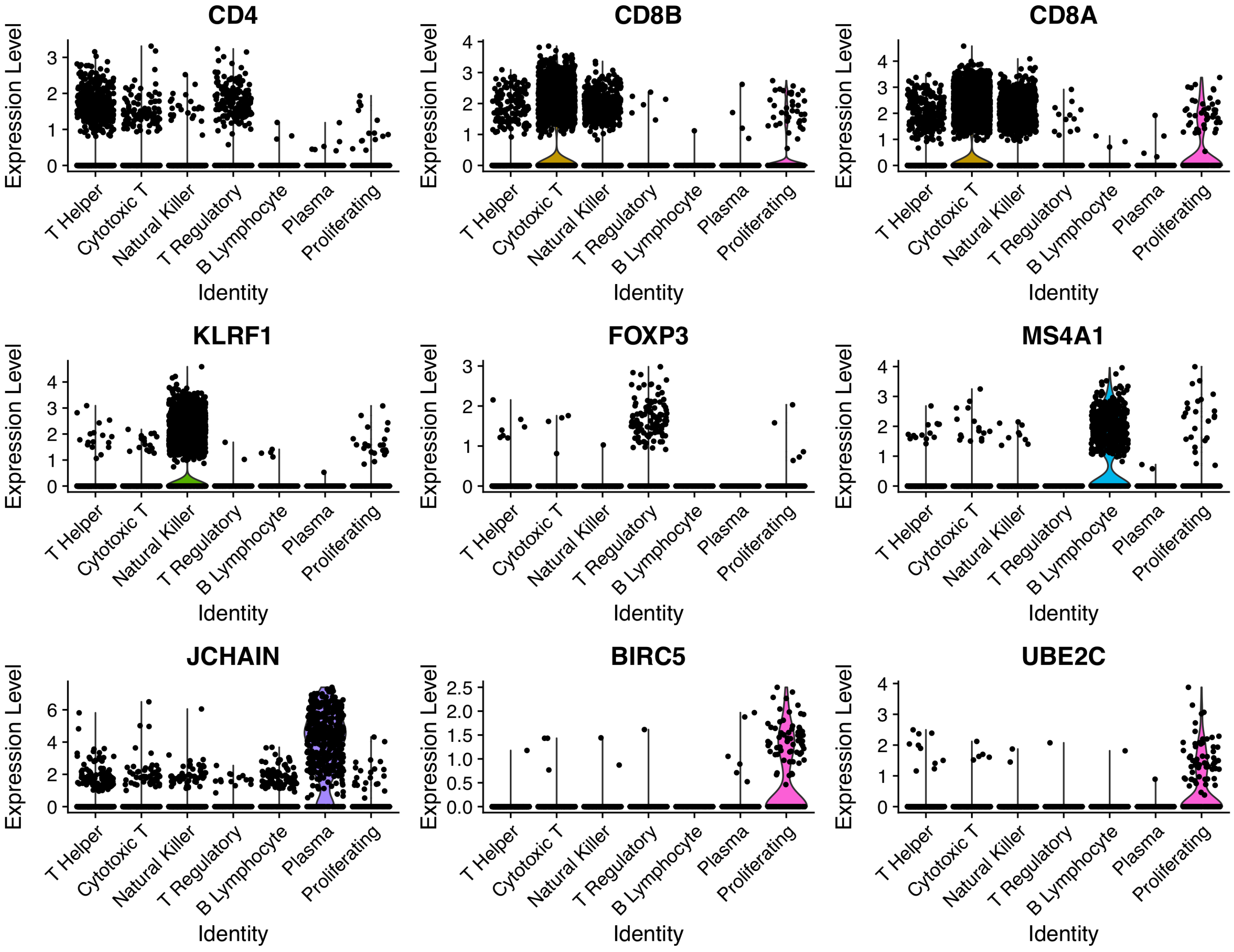

CD4
CD8B
CD8A
KLRF1
FOXP3
MS4A1
JCHAIN
BIRC5
UBE2C
Expression Level
Identity
T Helper
Cytotoxic T
Natural Killer
T Regulatory
B Lymphocyte
Plasma
Proliferating

Supplemental Figure 9. (A) Heat map of cytotoxic T cells, labeled by disease status, displaying 50 differentially expressed genes for the comparison of IPF and SSc-ILD cells. (B) Heat map of natural killer cells, labeled by disease status, displaying 50 differentially expressed genes for the comparison of IPF and SSc-ILD cells.

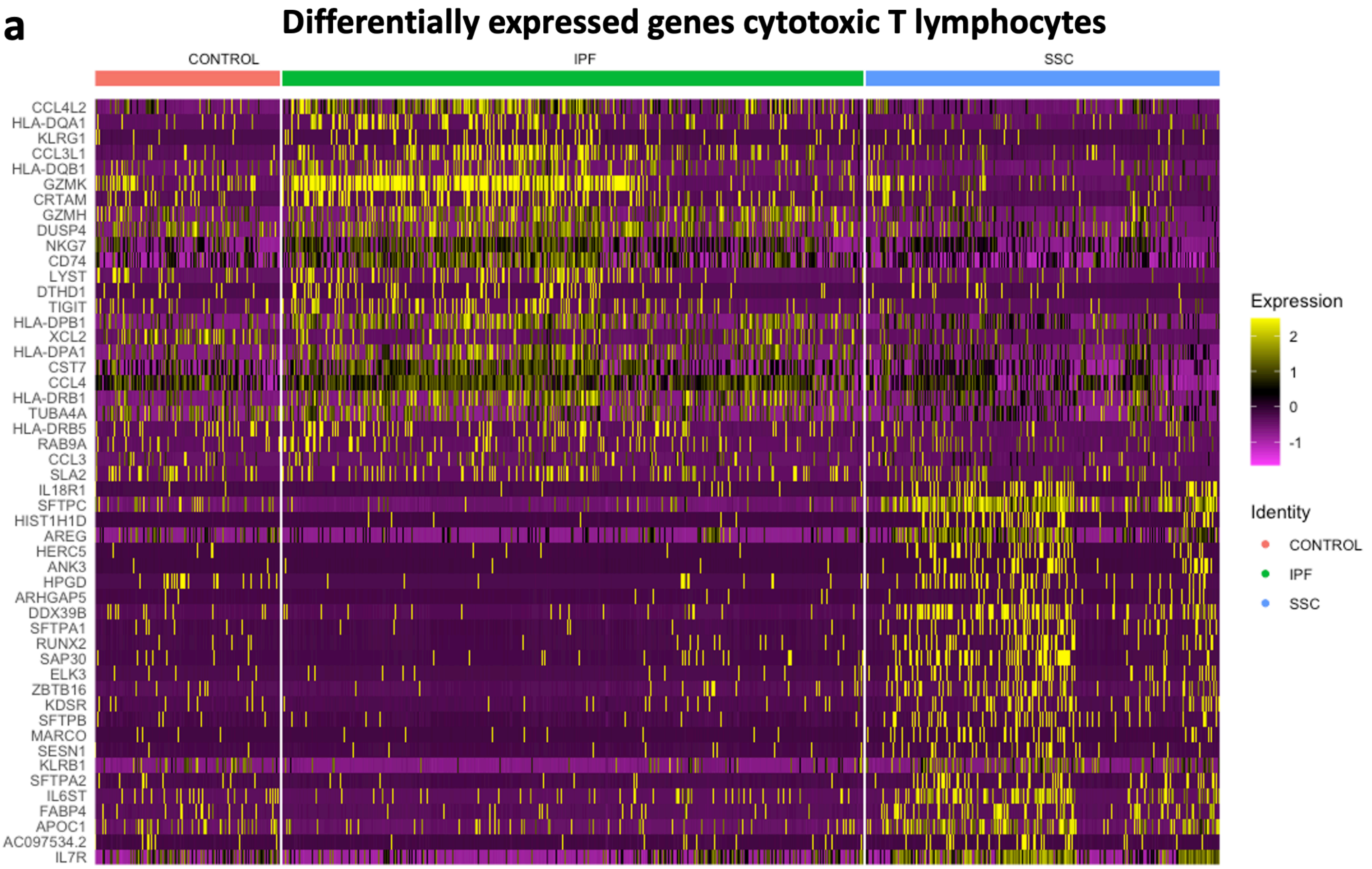
a
Differentially expressed genes cytotoxic T lymphocytes
CONTROL
IPF
SSC
CCL4L2
HLA-DQA1
KLRG1
CCL3L1
HLA-DQB1
GZMK
CRTAM
GZMH
DUSP4
NKG7
CD74
LYST
DTHD1
TIGIT
HLA-DPB1
XCL2
HLA-DPA1
CST7
CCL4
HLA-DRB1
TUBA4A
HLA-DRB5
RAB9A
CCL3
SLA2
IL18R1
SFTPC
HIST1H1D
AREG
HERC5
ANK3
HPGD
ARHGAP5
DDX39B
SFTPA1
RUNX2
SAP30
ELK3
ZBTB16
KDSR
SFTPB
MARCO
SESN1
KLRB1
SFTPA2
IL6ST
FABP4
APOC1
AC097534.2
IL7R
Expression
2
1
0
-1
Identity
CONTROL
IPF
SSC

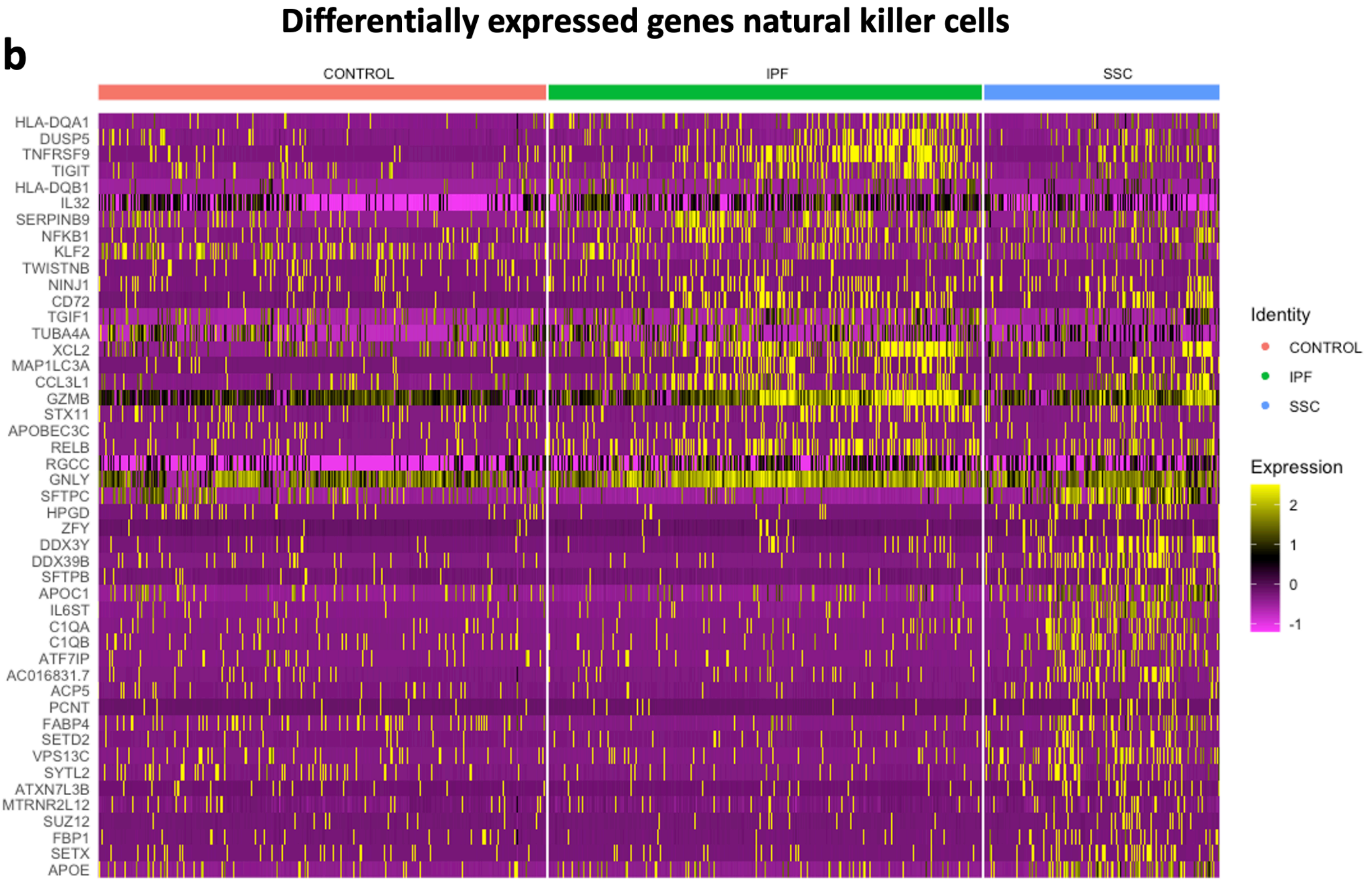
Differentially expressed genes natural killer cells
b
CONTROL
IPF
SSC
HLA-DQA1
DUSP5
TNFRSF9
TIGIT
HLA-DQB1
IL32
SERPINB9
NFKB1
KLF2
TWISTNB
NINJ1
CD72
TGIF1
TUBA4A
XCL2
MAP1LC3A
CCL3L1
GZMB
STX11
APOBEC3C
RELB
RGCC
GNLY
SFTPC
HPGD
ZFY
DDX3Y
DDX39B
SFTPB
APOC1
IL6ST
C1QA
C1QB
ATF7IP
AC016831.7
ACP5
PCNT
FABP4
SETD2
VPS13C
SYTL2
ATXN7L3B
MTRNR2L12
SUZ12
FBP1
SETX
APOE
Identity
CONTROL
IPF
SSC
Expression
2
1
0
-1

Supplemental Figure 10. (A) Heat map of plasmacytoid dendritic cells labeled by disease status, displaying 50 differentially expressed genes for the comparison of IPF and SSc-ILD cells. (B) Heat map of non-lymphatic endothelial cells, labeled by disease status, displaying 50 differentially expressed genes for the comparison of IPF and SSc-ILD cells.

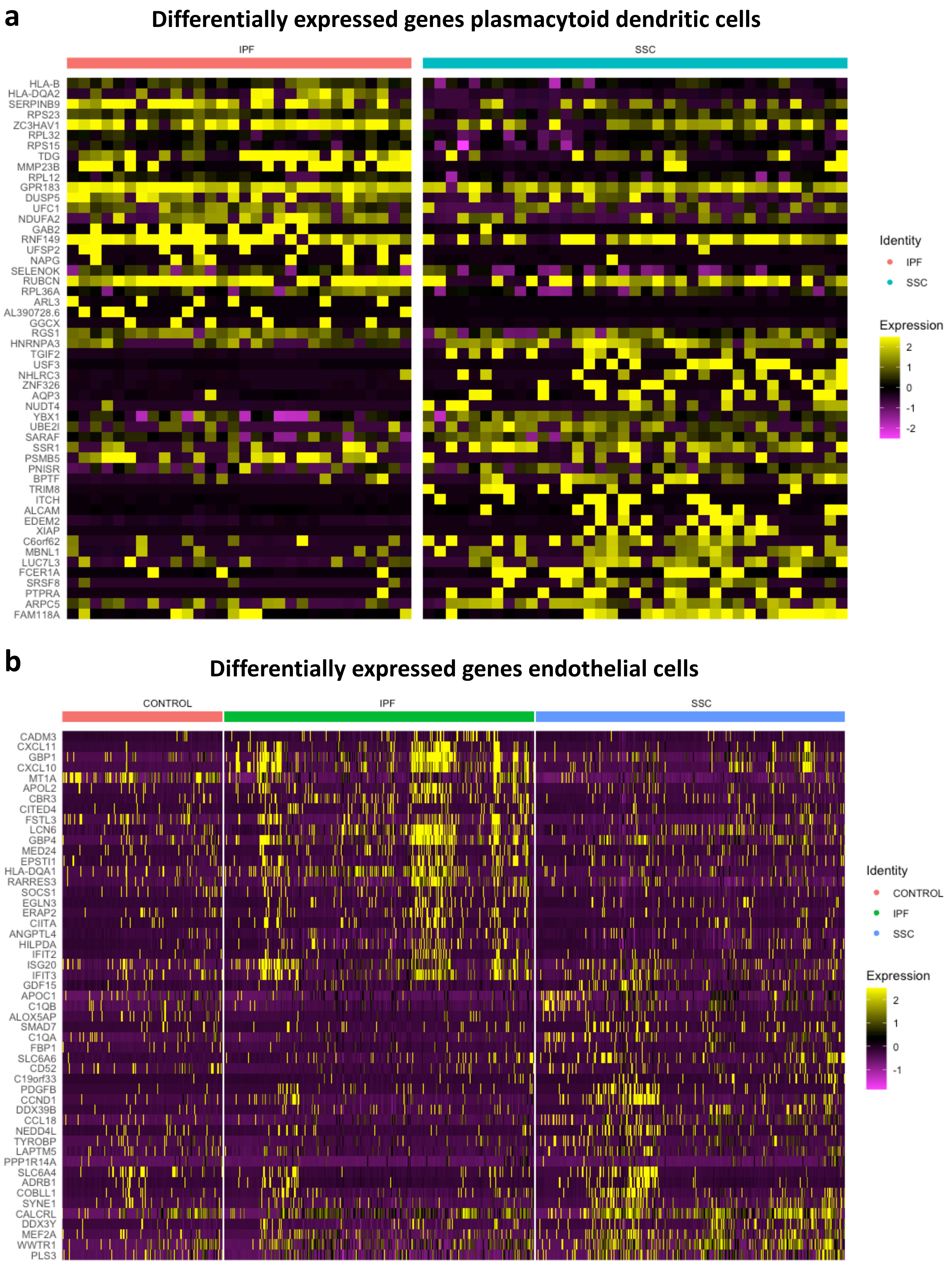
a
Differentially expressed genes plasmacytoid dendritic cells
IPF
SSC
HLA-B
HLA-DQA2
SERPINB9
RPS23
ZC3HAV1
RPL32
RPS15
TDG
MMP23B
RPL12
GPR183
DUSP5
UFC1
NDUFA2
GAB2
RNF149
UFSP2
NAPG
SELENOK
RUBCN
RPL36A
ARL3
AL390728.6
GGCX
RGS1
HNRNPA3
TGIF2
USF3
NHLRC3
ZNF326
AQP3
NUDT4
YBX1
UBE2I
SARAF
SSR1
PSMB5
PNISR
BPTF
TRIM8
ITCH
ALCAM
EDEM2
XIAP
C6orf62
MBNL1
LUC7L3
FCER1A
SRSF8
PTPRA
ARPC5
FAM118A
Identity
IPF
SSC
Expression
2
1
0
-1
-2
b
Differentially expressed genes endothelial cells
CONTROL
IPF
SSC
CADM3
CXCL11
GBP1
CXCL10
MT1A
APOL2
CBR3
CITED4
FSTL3
LCN6
GBP4
MED24
EPSTI1
HLA-DQA1
RARRES3
SOCS1
EGLN3
ERAP2
CIITA
ANGPTL4
HILPDA
IFIT2
ISG20
IFIT3
GDF15
APOC1
C1QB
ALOX5AP
SMAD7
C1QA
FBP1
SLC6A6
CD52
C19orf33
PDGFB
CCND1
DDX39B
CCL18
NEDD4L
TYROBP
LAPTM5
PPP1R14A
SLC6A4
ADRB1
COBLL1
SYNE1
CALCRL
DDX3Y
MEF2A
WWTR1
PLS3
Identity
CONTROL
IPF
SSC
Expression
2
1
0
-1

Supplemental Figure 11. ScRNA-seq analysis of IPF, SSc-ILD, and control epithelial populations with expression of selected cell type markers. Expression of *FOXJ1* indicates ciliated cells. Co-expression of *FOXJ1*, *MUC5B,* and *SCGB1A1* indicate secretory cells. Co-expression of *MUC5B* and *SCGB1A1* indicates goblet cells. Expression of *SCGB3A2* indicates club cells. Coexpression of *KRT5* and *KRT17* indicates basal cells. Expression of *KRT17* only indicates aberrant basaloid cells. Expression of *AGER* indicates AT1 cells. Expression of *SFTPC* indicates alveolar type 2 cells. Expression of *UBE2C* indicates proliferating cells. scRNA-seq, single-cell RNA-sequencing; IPF, idiopathic pulmonary fibrosis; SSc-ILD, systemic sclerosis-associated interstitial lung disease.

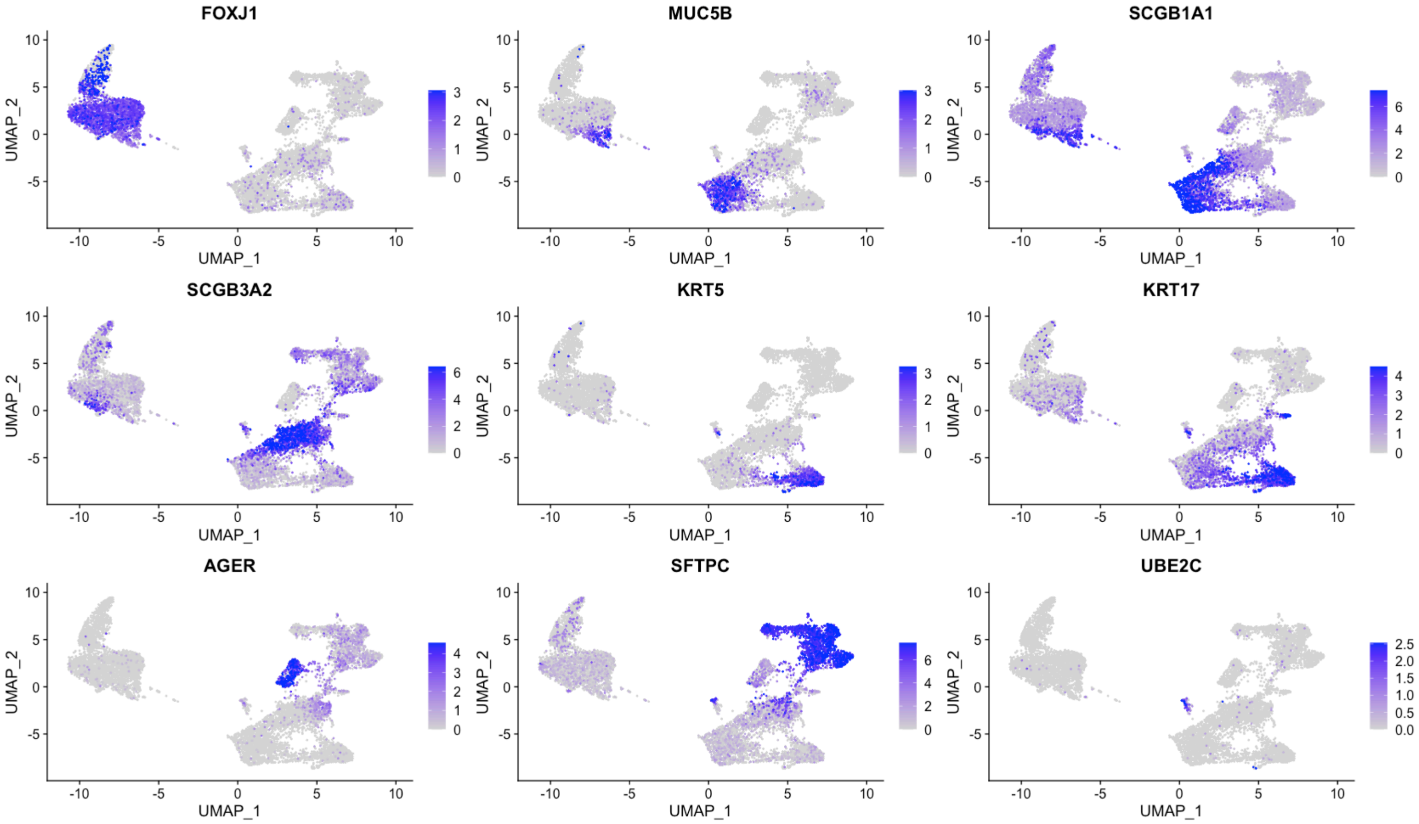

FOXJ1
MUC5B
SCGB1A1
SCGB3A2
KRT5
KRT17
AGER
SFTPC
UBE2C
UMAP_1
UMAP_2

Supplemental Figure 12. ScRNA-seq analysis of IPF, SSc-ILD, and control epithelial populations with expression of highlighted genes (or lack thereof in the case of *KRT5, KRT15,* and *SOX9*) in the aberrant basaloid cells. scRNA-seq, single-cell RNA-sequencing; IPF, idiopathic pulmonary fibrosis; SSc-ILD, systemic sclerosis-associated interstitial lung disease.

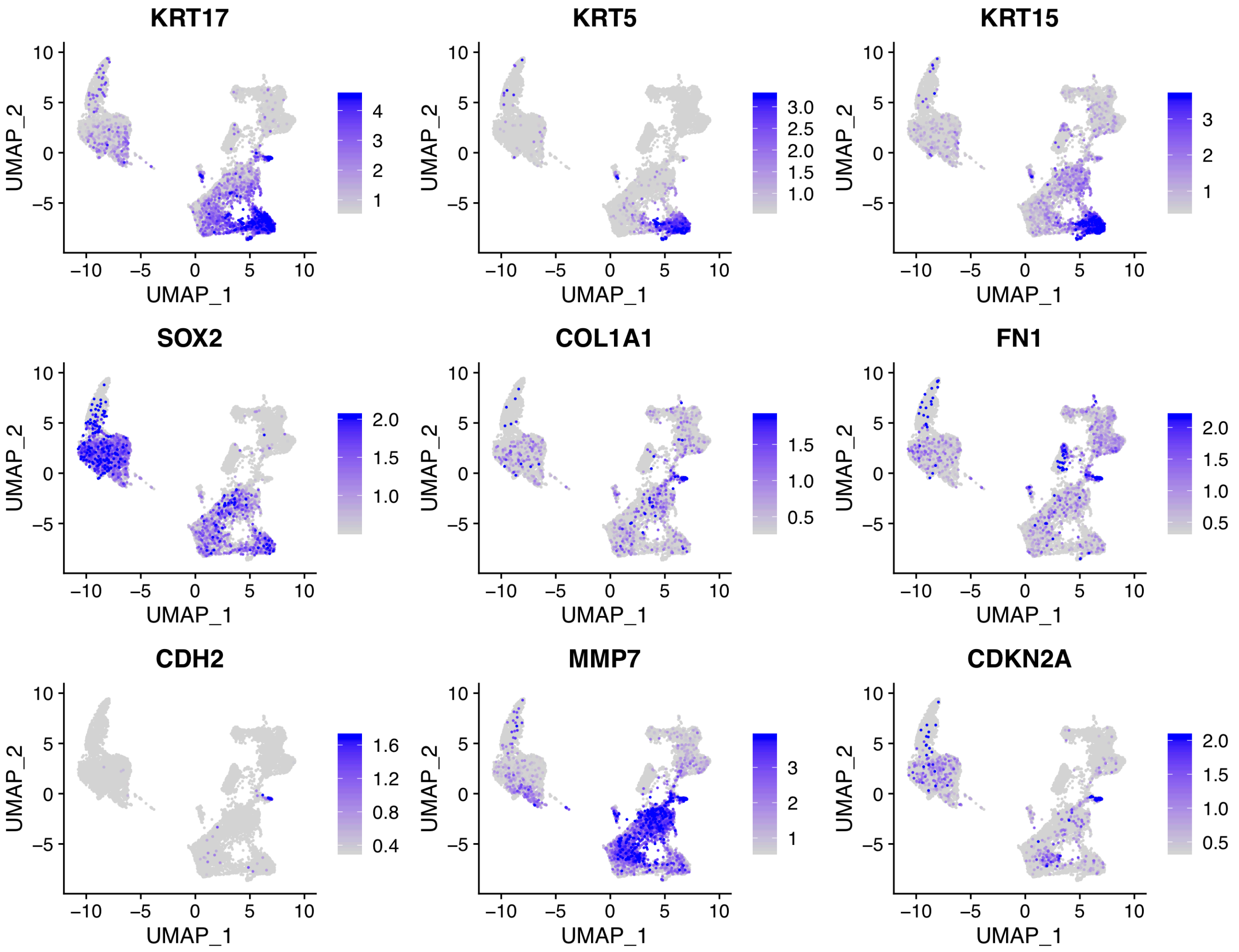
KRT17
KRT5
KRT15
SOX2
COL1A1
FN1
CDH2
MMP7
CDKN2A
UMAP_1
UMAP_2

Supplemental Figure 13. ScRNA-seq analysis of IPF, SSc-ILD, and control mesenchymal populations with expression of selected cell type markers. *LUM*- fibroblasts; *DES*- smooth muscle cells; *FAM162B*- pericytes; *ACTA2*-smooth muscle, pericyte, and myofibroblasts; *POSTN*-myofibroblasts and endothelial cells; *MFAP5*-*MFAP5*^{hi} fibroblasts; *SPINT2*-*SPINT2*^{hi} fibroblasts; *VWF*-endothelial cells; *CCL21*-lymphatic endothelial cells; *COL15A1*-peribronchial endothelial cells; *DKK2*-arterial endothelial cells; *HDAC9*-venous endothelial cells; *HPGD*-capillary endothelial cells; *UPK3B*-mesothelial cells; *TOP2A*-proliferating cells.
scRNA-seq, single-cell RNA-sequencing; IPF, idiopathic pulmonary fibrosis; SSc-ILD, systemic sclerosis-associated interstitial lung disease.

a

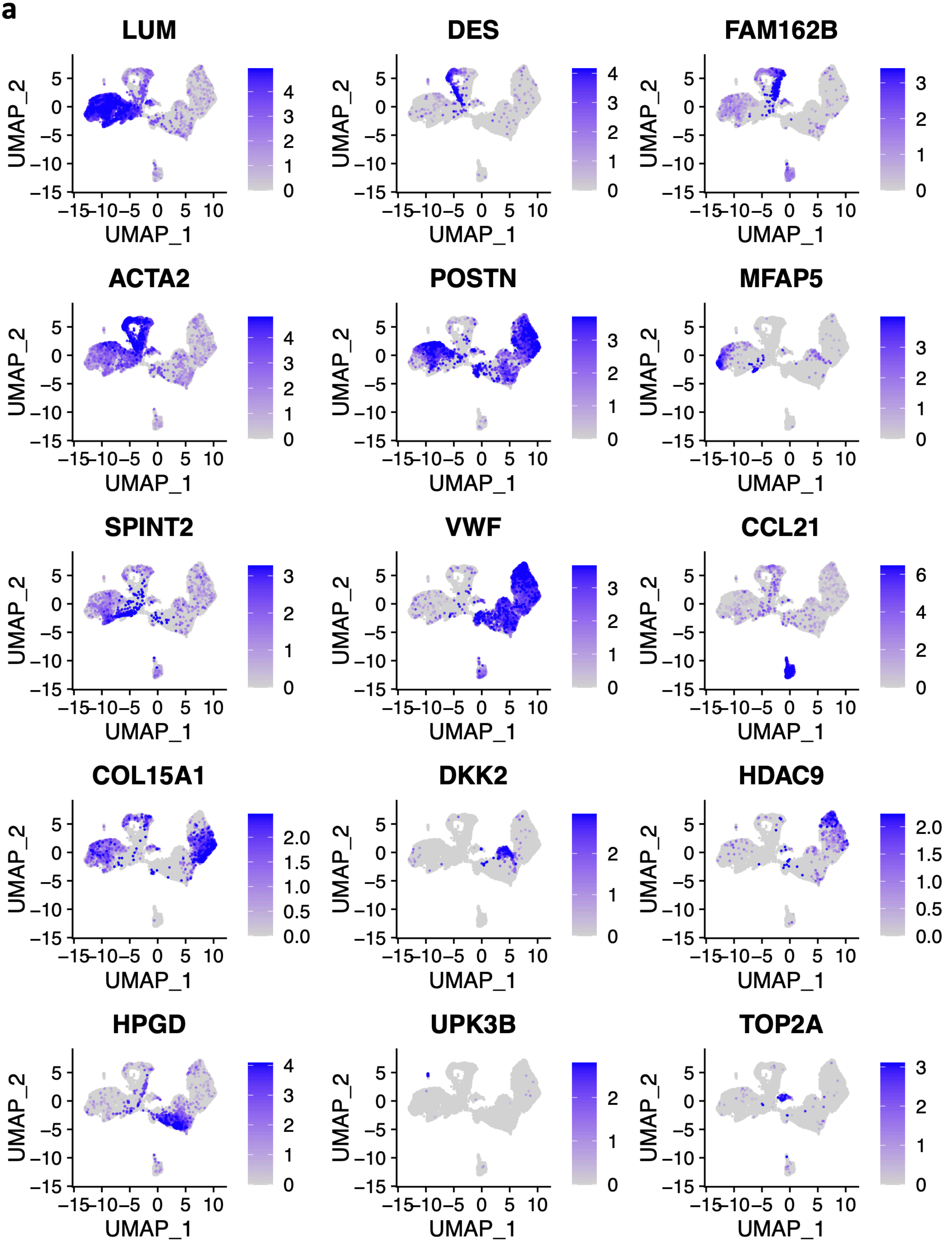
LUM
DES
FAM162B
ACTA2
POSTN
MFAP5
SPINT2
VWF
CCL21
COL15A1
DKK2
HDAC9
HPGD
UPK3B
TOP2A
UMAP_1
UMAP_2

Supplemental Figure 14. Gene ontology pathway analysis of endothelial cell subpopulations. (A) Pathways upregulated in IPF arterial endothelial cells compared to SSc-ILD arterial endothelial cells, and pathways upregulated in SSc-ILD arterial endothelial cells compared to IPF arterial endothelial cells. (B) Pathways upregulated in IPF capillary endothelial cells compared to SSc-ILD capillary endothelial cells, and pathways upregulated in SSc-ILD capillary endothelial cells compared to IPF capillary endothelial cells. (C) Pathways upregulated in IPF peribronchial endothelial cells compared to SSc-ILD peribronchial endothelial cells, and pathways upregulated in SSc-ILD peribronchial endothelial cells compared to IPF peribronchial endothelial cells. (D) Pathways upregulated in IPF venous endothelial cells compared to SSc-ILD venous endothelial cells, and pathways upregulated in SSc-ILD venous endothelial cells compared to IPF venous endothelial cells. If fold enrichment was calculated as >100 it is depicted as 100 for visualization.

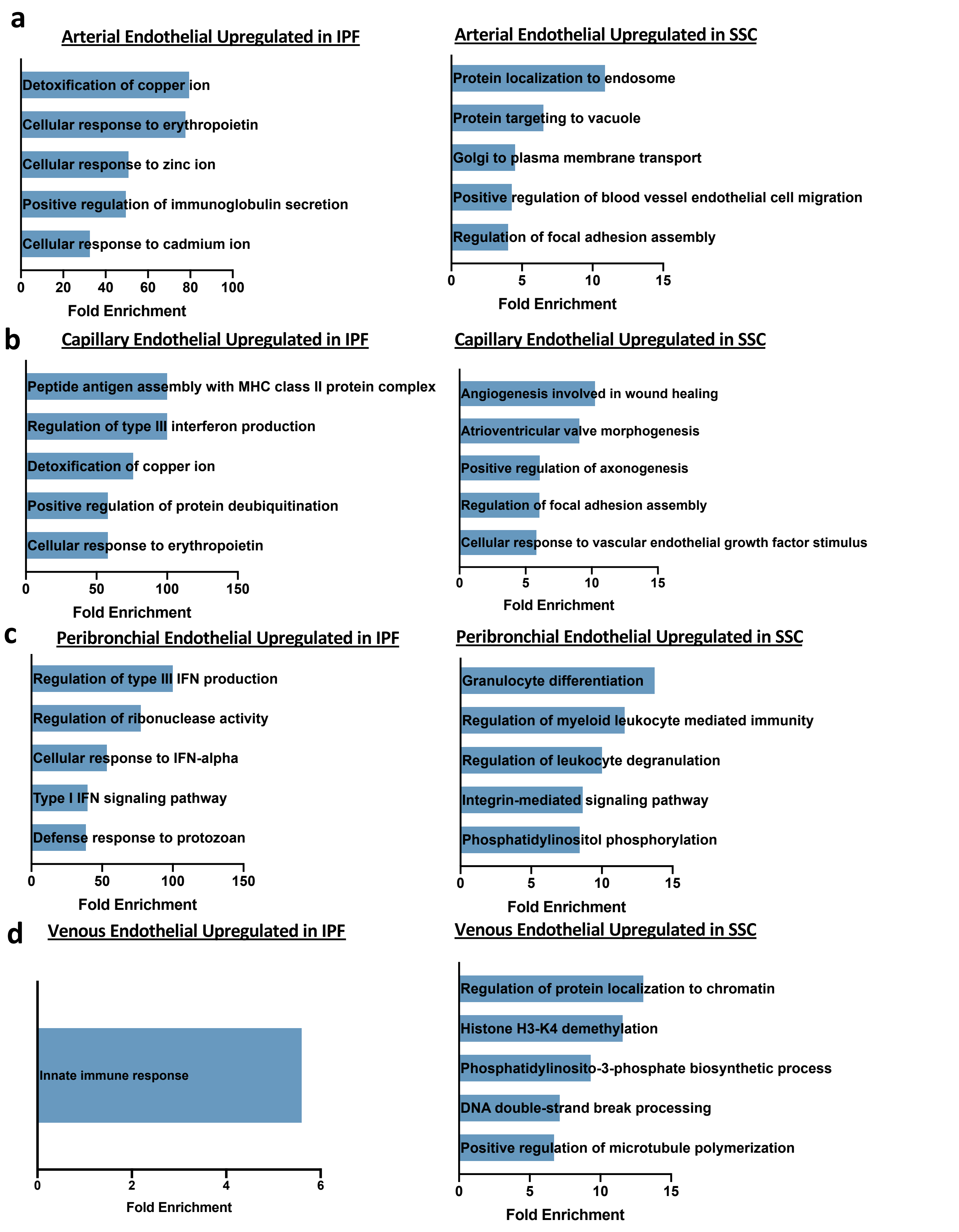
a
Arterial Endothelial Upregulated in IPF
Detoxification of copper ion
Cellular response to erythropoietin
Cellular response to zinc ion
Positive regulation of immunoglobulin secretion
Cellular response to cadmium ion
0 20 40 60 80 100
Fold Enrichment
Arterial Endothelial Upregulated in SSC
Protein localization to endosome
Protein targeting to vacuole
Golgi to plasma membrane transport
Positive regulation of blood vessel endothelial cell migration
Regulation of focal adhesion assembly
0 5 10 15
Fold Enrichment
b
Capillary Endothelial Upregulated in IPF
Peptide antigen assembly with MHC class II protein complex
Regulation of type III interferon production
Detoxification of copper ion
Positive regulation of protein deubiquitination
Cellular response to erythropoietin
0 50 100 150
Fold Enrichment
Capillary Endothelial Upregulated in SSC
Angiogenesis involved in wound healing
Atrioventricular valve morphogenesis
Positive regulation of axonogenesis
Regulation of focal adhesion assembly
Cellular response to vascular endothelial growth factor stimulus
0 5 10 15
Fold Enrichment
c
Peribronchial Endothelial Upregulated in IPF
Regulation of type III IFN production
Regulation of ribonuclease activity
Cellular response to IFN-alpha
Type I IFN signaling pathway
Defense response to protozoan
0 50 100 150
Fold Enrichment
Peribronchial Endothelial Upregulated in SSC
Granulocyte differentiation
Regulation of myeloid leukocyte mediated immunity
Regulation of leukocyte degranulation
Integrin-mediated signaling pathway
Phosphatidylinositol phosphorylation
0 5 10 15
Fold Enrichment
d
Venous Endothelial Upregulated in IPF
Innate immune response
0 2 4 6
Fold Enrichment
Venous Endothelial Upregulated in SSC
Regulation of protein localization to chromatin
Histone H3-K4 demethylation
Phosphatidylinosito-3-phosphate biosynthetic process
DNA double-strand break processing
Positive regulation of microtubule polymerization
0 5 10 15
Fold Enrichment

Supplemental Figure 15. Gene ontology pathway analysis of lymphatic endothelial cell subpopulation. (A) Pathways upregulated in IPF lymphatic endothelial cells compared to SSc-ILD lymphatic endothelial cells. (B) Pathways upregulated in SSc-ILD lymphatic endothelial cells compared to IPF lymphatic endothelial cells.

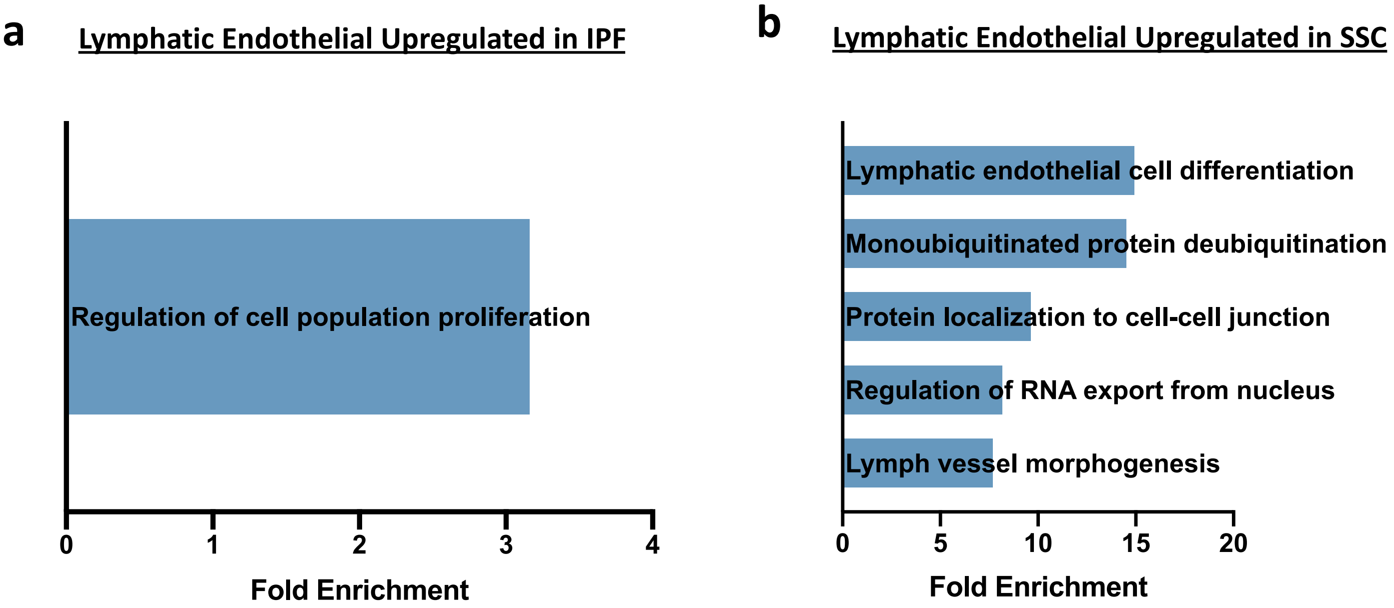
a
Lymphatic Endothelial Upregulated in IPF
Regulation of cell population proliferation
0
1
2
3
4
Fold Enrichment
b
Lymphatic Endothelial Upregulated in SSC
Lymphatic endothelial cell differentiation
Monoubiquitinated protein deubiquitination
Protein localization to cell-cell junction
Regulation of RNA export from nucleus
Lymph vessel morphogenesis
0
5
10
15
20
Fold Enrichment

Supplemental Figure 16. ScRNA-seq analysis of epithelial cell subpopulations with and without regression of cell phase when scaling data, demonstrating that controlling for cell phase does not alter the overall data's architecture. (A) UMAP plot of epithelial cells without (B) and with correction for cell phase labeled by cell type. (C) UMAP plot of epithelial cells without (D) and with correction for cell phase labeled by predicted cell phase. (E) UMAP plot of epithelial cells without (F) and with correction for cell phase labeled by disease status.

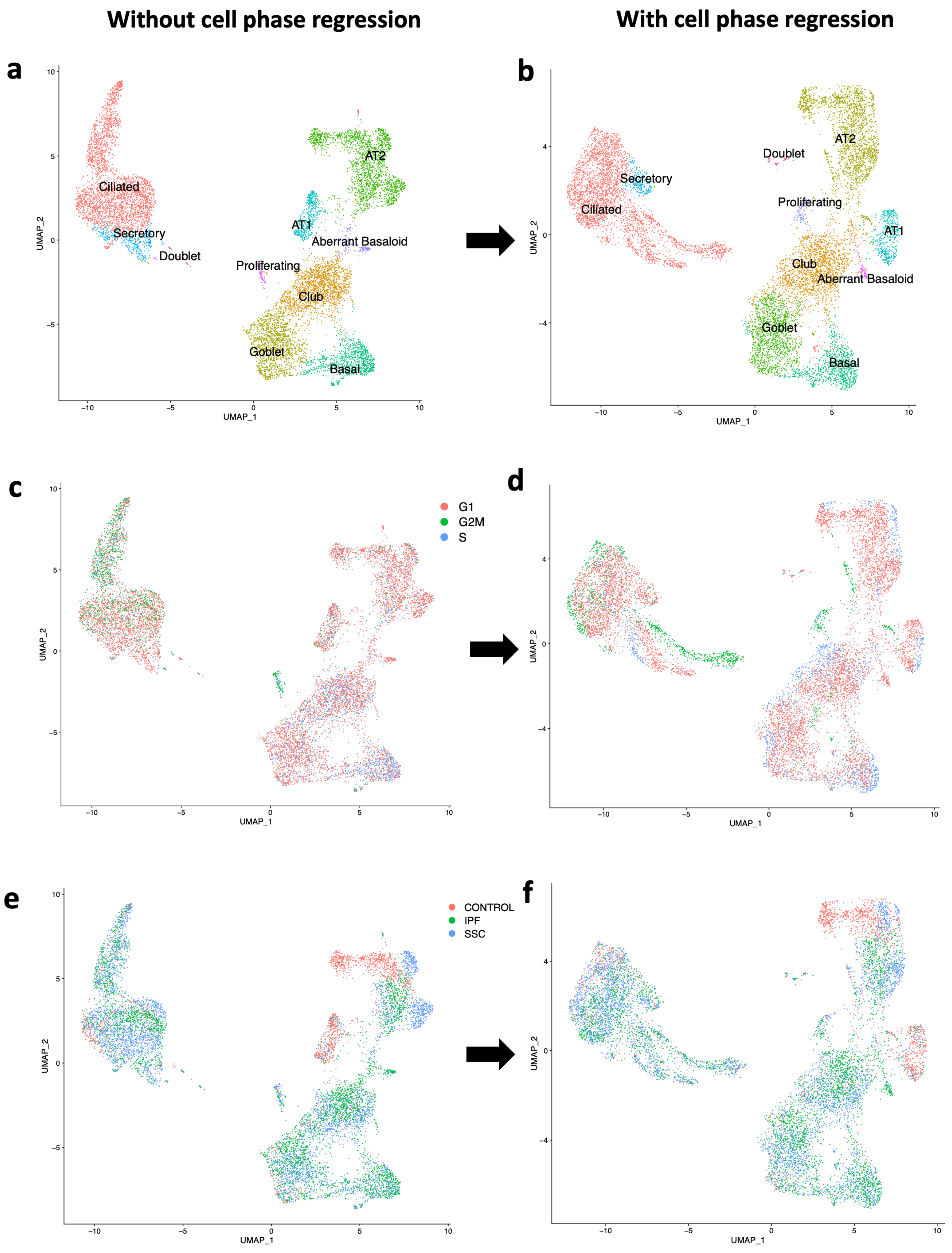
Without cell phase regression
With cell phase regression
a
b
c
d
e
f
Ciliated
Secretory
Doublet
Proliferating
AT1
AT2
Aberrant Basaloid
Club
Goblet
Basal
G1
G2M
S
CONTROL
IPF
SSC
UMAP_1
UMAP_2
